# Early transcriptional responses reveal cell type-specific vulnerability and neuroprotective mechanisms in the neonatal ischemic hippocampus

**DOI:** 10.1186/s40478-025-02062-4

**Published:** 2025-07-05

**Authors:** Aleksandr Ianevski, María Cámara-Quílez, Wei Wang, Rajikala Suganthan, Gunn Hildrestrand, Jonas Viken Grini, Dagny Sanden Døskeland, Jing Ye, Magnar Bjørås

**Affiliations:** 1 Department of Clinical and Molecular Medicine (IKOM), Norwegian University of Science and Technology (NTNU), 7491 Trondheim, Norway; 2 Department of Microbiology, Oslo University Hospital, University of Oslo, 0424 Oslo, Norway; 3 Centre for Embryology and Healthy Development, University of Oslo, 0373 Oslo, Norway

**Keywords:** Neonatal hypoxia ischemia, Single-cell RNA sequencing, Hippocampal cell atlas, Machine learning, Neural vulnerability, Gene regulatory network

## Abstract

Neonatal hypoxic-ischemic (H-I) brain injury, a leading cause of neurodevelopmental disabilities, severely affects the metabolically active and neurogenic hippocampus. To investigate its acute effects and identify drug targets for early therapeutic windows, we applied single-nucleus RNA sequencing on postnatal day 8 (P8) mouse hippocampi under sham, hypoxic, and hypoxic-ischemic conditions. We constructed a comprehensive hippocampal cell atlas and developed a machine-learning classifier for precise cell type identification. Our analysis reveals early vulnerabilities in mature neurons and notable resilience in immature DG, GABAergic, and Cajal-Retzius cells following H-I. Gene regulatory network analysis identified key transcription factors associated with neuronal vulnerability, along with upregulated ribosome biogenesis and dysregulated calcium homeostasis pathways. We observed rapid activation of astrocytes and microglia, with Runx1 identified as a potential key transcription factor associated with early microglia immune responses. Endothelial cells displayed complex transcriptional changes and predicted intercellular signaling patterns that may influence vascular repair and recovery. Our study advances the understanding of immediate cellular and transcriptional responses to neonatal H-I injury, providing new insights into hippocampal cell heterogeneity and pathophysiology. The integrated hippocampal atlas, post-H-I atlas, and machine learning classifier are available at https://hippo-seq.org.

**Supplementary Information:**

The online version contains supplementary material available at 10.1186/s40478-025-02062-4.

## Introduction

Hypoxia (oxygen deprivation) and ischemia (restricted blood flow) are severe conditions that disrupt cellular and molecular functions, critically affecting brain health [3, 29, 73]. In the neonatal period, the brain is particularly susceptible to hypoxic-ischemic (H-I) injury due to its high metabolic demands, ongoing developmental processes, and unique vulnerabilities such as elevated levels of unsaturated fatty acids, a high rate of oxygen consumption, and low concentrations of antioxidants [19, 74]. H-I brain injury remains a leading cause of neonatal mortality and long-term neurological disabilities, including cerebral palsy, epilepsy, and cognitive impairments [37, 41, 54, 75]. Among various brain regions, the hippocampus is especially vulnerable to H-I injury because of its high metabolic activity, extensive synaptic connectivity, and active neurogenesis during early postnatal development [15, 19, 47, 90].

Despite advancements in neonatal care, effective therapies to prevent or mitigate H-I-induced hippocampal damage remain limited [68, 75]. This is partly due to an incomplete understanding of the immediate cellular and molecular mechanisms underlying neuronal vulnerability and survival following H-I injury. Most previous studies have focused on delayed neuronal death and glial activation occurring days to weeks after the insult [7, 20, 24, 32]. While informative, these studies may overlook critical early events that determine the trajectory of injury and recovery. Understanding the acute responses that occur within hours of H-I injury is crucial, as this period represents a potential therapeutic window during which interventions could prevent irreversible damage and improve neurological outcomes [22, 43, 77, 91]. Elucidating the cell-type-specific molecular changes during this early phase can reveal key regulatory mechanisms and potential targets for neuroprotective strategies.

Various animal models of neonatal brain injury have been developed to explore cellular and molecular mechanisms and characterize functional outcomes of H-I, each with its own advantages and limitations [23]. In this context, our lab developed a modified Vannucci procedure to induce hypoxia–ischemia in postnatal day 8 (P8, DOB = P0) mice, consistently generating reliable injury outcomes [34, 48, 55, 60]. The P8 developmental stage, comparable to the human fetal period of 32–42 weeks of gestation, was selected for its dynamic balance between neurogenesis and gliogenesis, making it an ideal model for studying the acute effects of hypoxia–ischemia on these processes.

Single-nucleus RNA sequencing (snRNA-seq) has emerged as a powerful tool for dissecting cellular diversity in complex tissues, including brain [85]. Unlike single-cell RNA sequencing (scRNA-seq), which requires dissociating intact cells—a process particularly challenging for neurons—snRNA-seq isolates nuclei, making it suitable for profiling hard-to-dissociate fresh/frozen tissues as well as cryopreserved clinical samples [4, 38, 63]. This approach enables the characterization of cell-type-specific transcriptional responses at single-cell resolution in both healthy and diseased states [12, 63]. To investigate the immediate cellular and molecular adaptations in the neonatal hippocampus following H-I brain injury, we isolated nuclei from the p8 mouse hippocampus under sham, hypoxia-only, and hypoxia–ischemia conditions, and applied snRNA-seq using split-pool barcoding technology [56]. This method involves nuclei fixation followed by three rounds of barcoding, allowing high-resolution gene expression profiling from individual nuclei across multiple samples in a single experiment. The combinatory barcoding approach increases throughput and minimizes cost and batch effects, making it well-suited for studying complex tissues and comparing multiple conditions.

Accurately annotating cell types in snRNA-seq data is challenging, especially in the hippocampus (HPC), due to its intricate cellular diversity [86, 89] and the limitations of current computational methods. Existing annotation methods often struggle to resolve closely related cell types or identify rare populations even in healthy cell types [1, 16, 89], limiting the ability to detect subtle yet critical alterations in more challenging disease contexts. To overcome these challenges, we constructed a comprehensive hippocampal cell atlas by integrating publicly available single-cell transcriptomic datasets, encompassing over 420,000 cells across 33 distinct cell types. Leveraging this atlas, we developed a robust machine learning-based classifier for precise cell-type identification in both normal and pathological hippocampi. We validated our classifier by applying it to four recently published hippocampal transcriptomic datasets not used in its training, demonstrating enhanced cell-type annotations and correcting misclassifications.

We analyzed our snRNA-seq data and applied this classifier for accurate cell type annotation across sham, hypoxia-only, and post-H-I conditions. The analysis revealed specific vulnerabilities in mature neuronal types within the hippocampal CA1, CA3, and dentate gyrus (DG) regions as early as three hours post-H-I, while immature DG granule cells, GABAergic neurons, and Cajal-Retzius cells exhibited remarkable resilience. Gene regulatory network analysis identified key transcription factors associated with neuronal vulnerability, such as Hivep2, Bcl11a, Bcl6, and Thra, which are implicated in regulating calcium homeostasis and synaptic function. Concurrently, we observed rapid activation of astrocytes and microglia, marked by the upregulation of neuroinflammation and tissue repair genes, with Runx1 identified as a potential key regulator in microglia that is associated with early immune responses following ischemia. Endothelial cells exhibited a complex transcriptional response and altered intercellular signaling, potentially influencing vascular repair and modulating neuroprotection, neuroinflammation, tissue remodeling, and ischemic recovery.

Our study advances the understanding of immediate cellular and transcriptional responses to neonatal hypoxia–ischemia injury, providing new insights into hippocampal cell heterogeneity and pathophysiology, which facilitate the identification of potential drug targets and the development of neuroprotective strategies during this critical therapeutic window. All data—including the hippocampal atlas, post-H-I atlas, and the machine learning classifier—are publicly accessible via an interactive web application (https://hippo-seq.org).

## Materials and methods

### Animals

Mice with C57BL6NRj (Janvier labs, France) background were bred and housed in a 12-h light and dark cycle with food and water ad libitum. All experiments were approved by the Norwegian Animal Research Authority and conducted following laws and regulations controlling experimental procedures in live animals in Norway and the European Union Directive 86/609/EEC and experiments conducted in Brazil were approved by the Animal Ethics Committee of the Federal University of Rio Grande do Norte (CEUA Proj. No. 051/2015). For the snRNA-seq experiments, both male and female mice were used (Sham 1, Hypoxia 2, and H-I 2 were females; Sham 2, Hypoxia 1, and H-I 1 were males).

### Perinatal hypoxia–ischemia

Cerebral hypoxia and ischemia were produced in P8 (DOB = P0) mice by permanent occlusion of the left common carotid artery (CCA) followed by systemic hypoxia, as previously described^1^, with some modifications. In brief, pups were anesthetized with isoflurane (4% induction in chamber, 2.5% maintenance via mask with a 2:1 mixture of ambient air and oxygen). A midline incision was made on the ventral side of the neck, and the common carotid was carefully identified and separated from the vagus nerve. The artery was electrocoagulated using a monopolar cauterizer (Hyfrecator 2000; ConMed) set to 4.0 W. The neck incision was then closed with absorbable sutures (Safil 8–0 DRM6; B. Braun Melsungen AG). The surgical procedure was completed within 5 min. After the mice regained consciousness following surgery, they were returned to their dam in the cage for a 1-h recovery period. After 1-h recovery, the pups were exposed to a hypoxic (10% oxygen balance nitrogen; Yara), humidified atmosphere for 45 min at 36.0 °C. Afterward, the pups were returned to the cage with their dam and sacrificed 3 h post-hypoxia–ischemia. The ipsilateral brain hemisphere affected by carotid occlusion experiences hypoxic-ischemic conditions, while the contralateral brain hemisphere is exposed to hypoxia alone. Sham-operated animals were subjected to anesthesia and skin incision but not to occlusion of the CCA or hypoxia. Sham samples were taken from the left hippocampus, as the left hemisphere was experimentally manipulated for ischemia. All animals were sacrificed at equivalent time points (about five hours post-surgery intervention).

### Hippocampal nuclei isolation and fixation

Mice were sacrificed by decapitation and brains were excised. Hippocampi were isolated, snap-frozen in liquid nitrogen, and stored at − 70 °C freezer for further processing. Hippocampi were homogenized using a 1 ml glass Dounce Tissue Grinder (Wheaton, VWR, Cat. No. #62,400–595) in 1 ml of EZ lysis buffer (Sigma Aldrich, Cat. No. #N3408), with 10 strokes each using both Loose and Tight pestles for optimal nuclei extraction. The homogenate was filtered through MACS® SmartStrainers (30 µm) (Miltenyi Biotec, Cat. No. #130-098-458) and nuclei were pelleted by centrifugation (850xg for 10 min at 4 °C). The resulting pellet was resuspended in 2 ml of EZ lysis buffer and centrifuged again. Finally, the nuclei pellet was resuspended in 2 ml of PBS containing 1% BSA, and pelleted at 450xg for 10 min at 4 °C.

One million nuclei per sample were fixed using Evercode™ Nuclei Fixation v2 Kit from Parse Biosciences (cat. # ECF2103), following the manufacturer’s protocol. Briefly, nuclei were resuspended and incubated in the fixation solution for 10 min on ice, followed by a 3-min permeabilization on ice. The reaction was quenched with a Neutralization Buffer. Nuclei were then pelleted by centrifugation (350xg, 10 min, 4 °C) and resuspended in Nuclei Buffer (Parse Biosciences cat. #WN101) for a final count. DMSO (Parse Biosciences cat. #WN105) was added before freezing fixed nuclei at − 80 °C.

### Single-nucleus RNA sequencing (snRNA-seq) using split-pool combinatory barcoding technology

snRNA-seq was performed by Zenit Science https://zenitscience.com/single-cell-transcriptomics. Briefly, fixed nuclei samples prepared using the Parse protocol were rapidly thawed in a 37 °C water bath and immediately placed on ice. Barcoding of single nuclei, amplification of barcoded cDNA, and preparation of cDNA libraries for sequencing were performed using Evercode™ WT v2 Kit from Parse Biosciences (cat. #EC-W02030) following the manufacturer’s protocol. Briefly, individual nuclear transcriptomes were uniquely labeled by passing fixed nuclei through four rounds of barcoding. In the 1 st round of barcoding, cDNA was generated with in-nucleus reverse transcription (RT) reactions using well-specified barcoded primers. After RT, nuclei were pooled and distributed in 96 wells for the 2nd round of barcoding by in-nucleus ligation. After Round 2, nuclei were pooled and redistributed again into 96 wells for the 3rd round of barcoding by in-nucleus ligation. After Round 3, nuclei were pooled and split into 8 distinct populations, termed sublibraries. The final split nuclei were lysed and the barcoded cDNA underwent template switching and amplification. The cDNA was cleaned using AMPure XP beads (Beckman Coulter cat. #A63880) and quality checked using the Qubit dsDNA HS Assay Kit (Thermo cat. #Q33231) and a Bioanalyzer 2100 High Sensitivity DNA Kit (Agilent cat. #5067-4626). Each cDNA sublibrary was fragmented and Illumina P5/P7 adapters were ligated during the final amplification (the 4th sublibrary-specific barcode), followed by size selection and quality check with the Bioanalyzer and Qubit. Libraries, with 5% PhiX spike-in, were sequenced on an Illumina NovaSeq 6000 S4 Flow Cell using 150 bp paired-end reads, achieving an average depth of 50,000 reads per nucleus.

### Construction of reference hippocampus cell atlas

To generate a comprehensive hippocampal cell (HCA) atlas, we integrated publicly available single-cell and single-nucleus RNA sequencing datasets from mice of various ages and experimental conditions, totaling 424,225 cells. The datasets included: (1) Drop-seq data from adult male mice aged P60–70 [58] (*n* = 151,966; GEO: GSE116470); (2) nuclear RNA-seq from young (5 months) and aged (24 months) mice under dietary restriction [46] (*n* = 90,450; GEO: GSE227515); (3) snRNA-seq from mice aged 10–16 weeks receiving electroconvulsive stimulation [85] (*n* = 13,200; GitHub: https://github.com/Erik-D-Nelson/ARG_HPC_snRNAseq); (4) scRNA-seq data from the Allen Brain Cell Atlas [86] (7–10 weeks old; *n* = 161,011; GEO: GSE246717); and (5) SMART-seq profiling of ~ 8-week-old wild-type mice [46] (*n* = 7598; available at https://portal.brain-map.org/atlases-and-data/rnaseq/mouse-whole-cortex-and-hippocampus-smart-seq). For dataset integration and annotation, we used scANVI [82], a semi-supervised variational autoencoder model designed for single-cell data. Each dataset was loaded into an AnnData object and preprocessed with Scanpy version 1.9.8 [80], including normalization and selection of the top 5,000 highly variable genes per batch. We registered the data with scVI [45] by specifying the counts layer and batch information, and initially trained a scVI model with a 50-dimensional latent space to capture the underlying gene expression patterns. We provided cell type labels only for cell types that had consistent names across datasets; otherwise, labels were kept as unlabeled_category ="Unknown". We then initialized the scANVI model from the pretrained scVI model and trained it for 20 epochs using both labeled and unlabeled data to improve cell type annotation. After training, clusters labeled as"Unknown"were manually examined, and cell type names were unified by majority vote within each cluster. The latent representations and cell type predictions were obtained using the get_latent_representation() and predict() functions, respectively. To visualize the integrated data, we applied UMAP dimensionality reduction to the latent space embeddings, enabling us to assess clustering and cell type distributions across datasets. The interactive version of integrated hippocampal cell atlas (HCA) atlas is implemented using PHP v7.2 and D3.js version 7 [8], and is available at https://hippo-seq.org/hca.

### Development of classifier for automated hippocampal cell type annotation

To develop a classifier that accurately annotates hippocampal cell types, we enhanced the ScType [28] cell type classification method and fine-tuned its accuracy specifically for hippocampal tissue. As a first step, we identified an optimal set of marker genes that allow for precise and specific annotation of each cell type within the hippocampus. This optimization was performed using the integrated hippocampal cell atlas (HCA) of 424,225 cells. However, there are over 10^83^ possible marker gene sets when selecting between 1 and 30 marker genes per cell type for 33 HCA cell types, rendering exhaustive search computationally infeasible and prone to overfitting to HCA. To efficiently navigate this vast search space, we employed advanced machine learning techniques, specifically a Bayesian optimization approach in combination with expectation maximization (EM) and a robust leave-one-cell-type-out (LOCTO) cross-validation strategy.

Our objective was to find the optimal set of marker genes $S^{*}$ that maximizes the classification accuracy $S^{*} = \mathop {\arg \max }\limits_{S} f(S)$, where *S* represents a candidate set of marker genes for all cell types. To model the relationship between marker gene sets and classification accuracy, we constructed a Gaussian Process (GP) surrogate model ${\mathcal{G}\mathcal{P}}(\mu (S),k(S,S^{\prime}))$, where $\mu (S)$ is the mean prediction of accuracy for gene set *S*, and $k(S,S^{\prime})$ is the kernel function measuring similarity between gene sets *S* and *S*′. At each iteration *t*, we selected the next candidate marker gene set $S_{t + 1}$ by maximizing the Expected Improvement (EI) acquisition function $S_{t + 1} = \arg \max_{S} {\text{EI}}(S) = \int_{ - \infty }^{\infty } {\max } (0,f - f_{{{\text{best}}}} )p(f|S){\kern 1pt} df$, where $f_{{{\text{best}}}}$ is the highest accuracy observed up to iteration *t* and $p(f|{\mathcal{S}})$ is the posterior distribution of the performance given *S*. Within each iteration, we used an Expectation Maximization (EM) algorithm where we first assigned cell types based on current marker genes, then updated these markers to maximize the likelihood of the observed cell type assignments. The Bayesian optimization modelling was implemented using mlrMBO v1.1.5.1 R package [6].

To evaluate the performance of each candidate marker gene set and prevent overfitting to HCA atlas, we implemented a Leave-One-Cell-Type-Out (LOCTO) cross-validation strategy combined with five-fold cross-validation. For each cell type *c ∈ {1,…,C}* (where *C* = 33), we temporarily excluded its marker genes from *S*, causing ScType to label cells of type *c* as"unknown". ScType was applied with the modified *S* to annotate the cells. For the fold *k*, we calculated the accuracy $f_{c,k} ({\mathcal{S}})$ as the proportion of correctly assigned cells among all cell types (including cells of type *c*). We repeated this process across all cell types and *K* = 5 data splits (folds), obtaining an overall accuracy: $f({\mathcal{S}}) = \frac{1}{C \times K}\sum\nolimits_{c = 1}^{C} {\sum\nolimits_{k = 1}^{K} {f_{c,k} } } ({\mathcal{S}})$.

To enhance computational efficiency, we developed a vectorized version of ScType. Given the input sc/nRNA-seq data $X \in {\mathbb{R}}^{m \times n}$ with *m* genes and *n* cells, ScType first standardizes each gene expression profile into z-scores across all cells and multiplies it with its marker specificity score *θ*_*i*_: $X^{\prime} = (Z(X^{{\text{T}}} ))_{{ \subseteq M_{t} }}^{{\text{T}}} \cdot \Theta$, where *X′* is the transformed scRNA-seq expression matrix of *n* cells and ∣*M*_*t*_∣ marker genes, within tissue *t* (in this case, hippocampus). The marker sensitivity scores *θ*_*i*_ are calculated based on the current candidate marker gene set *S*. Specifically, *θ*_*i*_ ​ reflects the specificity of gene *i* across the cell types in *S*: $\theta_{i} = \frac{{\frac{1}{{n_{i} }} - \min_{j} \left( {\frac{1}{{n_{j} }}} \right)}}{{\max_{j} \left( {\frac{1}{{n_{j} }}} \right) - \min_{j} \left( {\frac{1}{{n_{j} }}} \right)}}$, where *n*_*i*_​ is the number of cell types where gene *i* appears in *S*. Here, the rescaling maps *θ*_*i*_ to the range [0,1], ensuring that genes appearing in fewer cell types receive higher scores. Next, the standardized expressions of marker genes are multiplied by their specificity scores *θ*_*i*_: $\tilde{Z}_{i,j} = Z_{i,j} \times \theta_{i} ,\;\;\;{\kern 1pt} {\text{for }}i \in {\mathcal{S}}$. Finally, for each cell type *c* and cell *j*, the marker enrichment score *E*_*c,j*_ ​ is calculated as $E_{c,j} = \frac{1}{{\sqrt {|S_{c} |} }}\sum\nolimits_{{i \in S_{c} }} {\tilde{Z}_{i,j} }$, and cell type with the highest enrichment score $\arg \max_{c} E_{c,j}$ is set for cell *j*.

The surrogate model and refined marker genes were iteratively updated during 1,000,000 iterations and the optimal marker set *S*^*∗*^ was then used for cell type annotations with the vectorized ScType version.

We validated the optimized marker-based classifier on four independent hippocampal transcriptomic datasets. The datasets were downloaded from Single Cell Portal (SCP, https://singlecell.broadinstitute.org/single_cell)—SCP2162 [57], SCP1375 [87], SCP2065 [18] and SCP110 [21]. We normalized the raw count values of each dataset using Transcripts Per Million (TPM). The datasets were further processed using the Seurat v5.0.2 [25] standard workflow and UMAP visualization was performed with 20 principal component analysis (PCA) dimensions. Subsequently, we applied vectorized ScType with a machine-learning-optimized marker gene set to each of these datasets.

### Processing snRNAseq data

For single-nucleus RNA sequencing analysis, we utilized the Seurat v5.0.2 [25] R package. Following quality control filtering (minimum 200 genes per nucleus, maximum 20,000 nuclear UMIs/nUMIs), data was normalized using SCTransform and principal component analysis (PCA) was performed on the top 3000 variable genes. The first 30 principal components were used for uniform manifold approximation and projection (UMAP) dimensionality reduction and graph-based clustering with a resolution of 0.5. We utilized canonical correlation analysis (CCA) on the top 30 principal components with Seurat’s IntegrateLayers function, resulting in a unified dataset. Cell types were annotated using vectorized ScType with a machine-learning-optimized marker gene set. For differential expression analysis, we aggregated single-cell data into pseudobulk samples by averaging expression within each biological replicate (two mice per condition) and cell type using Seurat's AverageExpression function, thereby enhancing statistical power and mitigating false discovery rates [65]. Differential expression analysis was performed using the limma-voom pipeline from the limma version 3.56.2 package in R [53], utilizing pseudobulk samples, with sex included as a covariate in the design matrix to account for potential sex-specific effects. SCENIC (Single-Cell rEgulatory Network Inference and Clustering) analysis was performed using pySCENIC version 0.12.1 [2, 70] command line interface (CLI) within a Docker container downloaded from https://hub.docker.com/r/aertslab/pyscenic. The expression data were normalized using TMM normalization and a log1p transformation, followed by generating co-expression modules with the GRNBoost2 algorithm using ‘pyscenic grn’, separately for each condition. We then refined the networks using ‘pyscenic ctx’ with RcisTarget version 10 to retain only direct target genes with TF binding motifs, and finally applied AUCell to quantify regulator activity in each cell with ‘pyscenic aucell’. The pathway enrichment analysis employed clusterProfiler R package version 4.8.3 [81] to perform KEGG pathway and GO enrichment analyses on differentially expressed genes, with significance thresholds of adjusted *p* value less than 0.05, minimum gene count of 3, and gene ratio ≥ 5%. For network visualizations, igraph 1.5.1 R package [11] and D3.js version 7 were utilized. The interactive version of integrated post-hypoxia–ischemia cell atlas is implemented using PHP v7.2 and D3.js version 7, and is available at https://hippo-seq.org/hi.

### Paraffin brain sectioning and staining

Mice were deeply anesthetized with a single subcutaneous dose of fentanyl/fluanisone plus midazolam (ZRF) followed by transcardial perfusion with PBS. The mouse brains were excised and immersion-fixed in 4% paraformaldehyde for at least 24 h. The fixed brains were then dehydrated and hemisected along the midline, and both hemispheres were embedded side-by-side in the paraffin block. Sagittal brain Sects. (4 μm thick) of both hemispheres were sectioned using the microtome Leica RM2255 (Leica Biosystems) and mounted on SuperFrost Plus™ Adhesion slides (Epredia™, Thermo Fisher Scientific cat. # J1800AMNZ). The sections were allowed to dry in the oven at 37 °C overnight and stored at 4 °C for subsequent processing.

Sagittal brain Sects. (4 µm) were subjected to antigen retrieval in a buffer containing 40 mM trisodium citrate (pH 6.0) at 99 °C for 3 min and then washed with PBS. After blocking the sections in a blocking buffer (PBS with 5% normal goat serum, 1% BSA, and 0.1% Triton X-100) at room temperature for 1 h, the sections were incubated with primary antibodies in a dilution buffer (PBS with 1% normal goat serum, 1% BSA, and 0.1% Triton X-100) overnight at 4 °C. The next day, sections were washed three times with 1 × PBST and incubated with secondary antibodies in the same dilution buffer at room temperature for 1 h. After another three washes in 1 × PBST, sections were mounted on glass slides and left to dry overnight. Finally, sections were stained with DAPI (1 μg/mL in PBS), washed, and cover-slipped using ProLong™ Gold Antifade Mountant with DAPI (Thermo Fisher). The first antibodies used are NeuN (mouse IgG1 1:500, Merck Millipore, Cat. No. MAB377, RRID: AB_2298772), Calbindin1 (rabbit IgGs, 1:1000, Swant, Cat. No. CB 38a, RRID: AB_10000340), Calretinin (mouse IgG1, 1:1000, Swant, 7699/3H, RRID: AB_10000321). Secondary antibodies are from Thermo Fisher: Alexa Fluor 488 anti-rabbit (1:1000, Cat. No. A32731, RRID: AB_2633280) and Alexa Fluor 555 anti-mouse IgG1 (1:1000, Cat. No. A-21127, RRIDAB_2535769).

Tunel assay was performed using the In Situ Cell Death Detection Kit, Fluorescein following the manufacturer’s protocol (Roche, Cat. No. #11,684,795,910).

### 3D image analysis and statistics

Microscopy was carried out using a Zeiss LSM 880 confocal laser scanning microscope with a Plan-Apochromat 40x/1.4 Oil DIC M27 objective (Carl Zeiss, Jena, Germany). Z-stack images (1 µm intervals) of the entire hippocampus were captured for analysis. 3D nucleus segmentation and fluorescence intensity measurements were carried out within the nuclei. Briefly, Nuclei were segmented using the Stardist segmentation algorithm [78], with a custom model trained on DAPI-stained, 4 and 30-um thick mouse hippocampal images. The mean fluorescence intensity (MFI) of each labeled nucleus was measured using the scikit-image [71]. NeuN-positive cells were classified based on the mean NeuN fluorescence intensity within the nucleus. K-means clustering (three clusters) was applied to categorize nuclei into three groups: NeuN-, weak NeuN^+^, and strong NeuN^+^. The clustering algorithm determined threshold values by minimizing variance within each group, thereby assigning nuclei to one of the three categories according to NeuN expression levels. The hippocampal subregions (CA1, CA3, and DG) were manually annotated and cell densities for each subregion were calculated. The number of NeuN^−^, weak NeuN^+^, and strong NeuN^+^ cells was quantified in the hippocampus under sham, hypoxia-only, and hypoxia–ischemia conditions. The cell density was calculated by manually annotating the analysis area and determining its volume. For calbindin and calretinin-positive cells, clustering was performed using K-means clustering with two clusters, and classification was limited to nuclei in the dentate gyrus (DG). Apoptotic cells were analyzed using the TUNEL assay and the TUNEL-positive cells were identified based on an MFI threshold of 1.6 × that of all nuclei. For quantifying cytoplasmic markers, such as GFAP, CD86, the cytoplasmic signal was approximated by dilating the nuclear masks approximately 1 μm and assigning each voxel to the closest nucleus. The mean fluorescence intensity (MFI) of each labeled area was then measured. GFAP and CD86-positive cells were classified using k-means clustering with two clusters. Only cells within the hippocampal region were selected for analysis.

The n-value for any analysis is reported as both the number of brain sections analyzed and the number of independent animals from which these sections were derived. Specific n-values for different markers and experimental conditions are provided in the accompanying Table. For clarity, ‘Contra’ refers to the hypoxia-only (contralateral) condition, while ‘Ipsi’ denotes the hypoxic-ischemic (ipsilateral) condition. Images with substantial damage to the analyzed area, such as rifts and tears, were excluded before analysis. After quantification, outliers were detected and removed using the interquartile range method. Statistical significance between groups was determined using ANOVA, with Tukey’s HSD test applied for post-hoc comparisons.

**Table:** 

Marker	Sham		Contra		Ipsi		Total	
	Animals	Sections	Animals	Sections	Animals	Sections	Animals	Sections
TUNEL	3	4	3	4	3	6	6	14
CD86	2	5	3	4	3	6	5	15
GFAP	2	7	3	6	3	6	5	19
Calb1	3	11	5	12	4	8	8	31
Calr	2	9	5	9	5	10	7	28
NeuN	3	6	3	4	3	6	6	16

## Results

### Generating a comprehensive hippocampal atlas for accurate cell-type annotation via machine learning

**Fig. 1:**
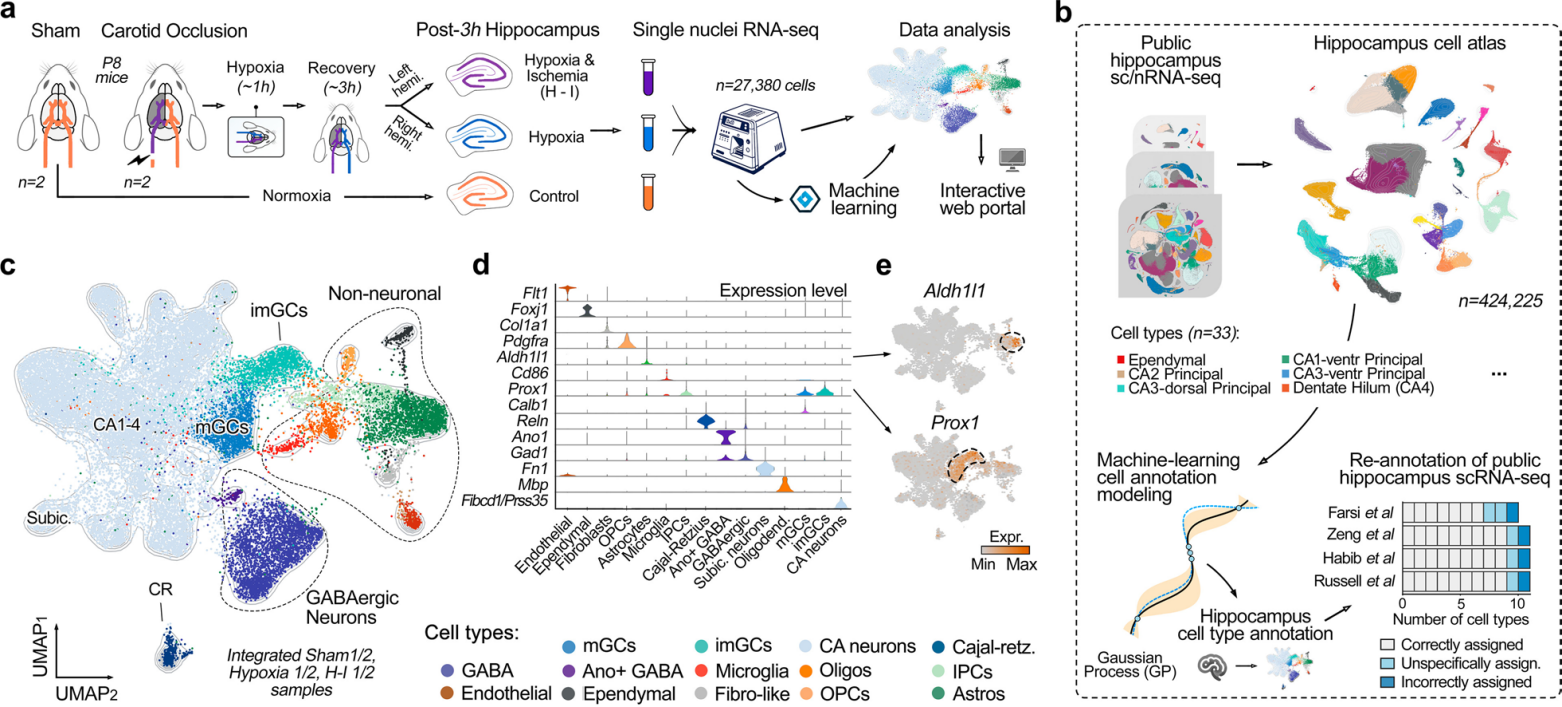
snRNA-seq analysis of early transcriptional changes in developing mouse hippocampus. **a** A schematic illustration of the experimental and analytical workflow. Micro-dissected hippocampal samples from postnatal day 8 (P8, DOB = P0) mice were subjected to three different conditions: sham, 45-min hypoxia, and 3-h post-H-I. Single-nucleus RNA sequencing (snRNA-seq) was performed on these samples, and the resulting data were analyzed to identify molecular and cellular changes. The processed data are available for exploration at the interactive web portal: https://hippo-seq.org. **b** Upper panels: Integration of publicly available scRNA-seq mouse hippocampus datasets. The right panel presents a UMAP visualization of the integrated reference hippocampus atlas (HCA) utilized in the machine learning model, which can be interactively explored at https://hippo-seq.org/hca. Lower panels: The left panel illustrates the machine-learning framework, where Gaussian process optimization was used to refine gene sets that define specific cell types (see methods). The optimized model was applied to annotate four independent public single-cell transcriptomic datasets (right panel), where it automatically assigned cell types as reported in the original studies, while reannotating five unspecifically classified and four incorrectly assigned cell types. **c** Integrated UMAP visualizations of all three hypoxia–ischemia conditions in P8 mice, with major cell types automatically annotated using the machine-learning-based model. An interactive version of the data is available at https://hippo-seq.org/hi. **d** Violin plots showing the expression levels of high-specificity canonical marker genes for each cell type. **e** UMAP visualizations for selected marker genes

To explore early cellular and transcriptional adaptations in the neonatal hippocampus following H-I, we induced cerebral ischemia in wild-type mice at postnatal day 8 (P8) by permanently occluding the common carotid artery in the ipsilateral brain hemisphere, followed by systemic hypoxia (Fig. 1a). Histological examinations of samples demonstrated significant injury in the cortex, hippocampus, striatum, and thalamus of the affected hemisphere, whereas the contralateral hemisphere remained largely undamaged, showing no visible harm when compared to the brains of sham-treated mice [60].

To further dissect the cellular alterations induced by H-I, we performed single-nuclei RNA sequencing (snRNA-seq) using the split-pool combinatorial barcoding technology (Parse Biosciences, see Methods) on micro-dissected hippocampal samples from perinatal mice subjected to different conditions: sham treatment, 45-min hypoxia, and 3-h post-hypoxia–ischemia (Fig. 1a, Suppl. Fig. 1). Given the complexity and heterogeneity of the hippocampal cellular landscape, accurately annotating cell types in snRNA-seq data is critical, particularly under conditions of hypoxia and ischemia, which significantly alter transcriptional profiles.

To address the challenge of cell type annotation in the hippocampus, we first established a robust foundation by constructing a comprehensive reference hippocampal atlas. This atlas was built using well-annotated, publicly available transcriptomic datasets from the hippocampus, encompassing over 420,000 cells across 33 distinct cell types (Fig. 1b upper panel, Suppl. Fig. 2, https://hippo-seq.org/hca) (see Methods). The integration of these datasets was facilitated by variational autoencoders, which effectively harmonized diverse datasets, and Gaussian Process (GP)-based optimization, which identified a robust set of marker genes with high discriminatory power for different cell types. The resulting model automatically assigns cell types to query cells based on their gene expression profiles (Fig. 1b left bottom panel) (see methods).

To validate the robustness and accuracy of our model, we applied it to four recently published hippocampal transcriptomic studies, including a spatial transcriptomics dataset [18, 21, 57, 87]. Notably, these test datasets were not part of the data used for model training. Our analysis revealed significant discrepancies in cell type annotations across the published datasets when compared to the model's predictions (Suppl. Fig. 3). For example, the model precisely classified CA3 neurons into dorsal (Iyd ^high^) and ventral (Nkd2^high^) subtypes, astrocytes into reactive (Gfap^high^) and resident types, and reclassified incorrectly annotated neuroblast granule cells into immature (Prox1^+^Calb1^low^) and mature (Prox1^+^Calb1^high^) granule cells in Farsi et al. study (Suppl. Fig. 3a). Similarly, in the spatial transcriptomics dataset of Russel et al.[57], the model accurately distinguished mature and immature granule cells and reannotated oligodendrocytes into oligodendrocytes (Mog^+^) and oligodendrocyte precursor cells (Pdgfra^+^) (Suppl. Fig. 3d, e). Altogether, among the four datasets, our model reannotated five unspecifically assigned and four incorrectly identified cell types (Fig. 1b, right bottom panel).

These findings underscore the limitations of current annotation methodologies in accurately characterizing the complex cellular architecture of the hippocampus and highlight the superior accuracy and specificity of our approach. Similarly, the use of supervised learning techniques has proven to be highly effective in distinguishing transcriptionally ambiguous cell subtypes across various single-cell datasets from multiple systems and disease contexts [64, 72, 89]. The implemented machine learning-based model for annotating user-provided single-cell hippocampus data, along with the integrated hippocampal cell atlas, is publicly accessible at https://hippo-seq.org.

### Cell census of the neonatal hippocampus under hypoxia and ischemia

Next, we applied the developed machine learning model to our snRNA-seq dataset, which consisted of 27,380 nuclei from six hippocampal samples—2 × sham-control, 2 × hypoxic, and 2 × hypoxic-ischemic. Unsupervised clustering based on global gene expression profiles, followed by UMAP visualization for dimensionality reduction, revealed fourteen distinct cell populations (Fig. 1c). The pre-trained model was then employed to assign cell types to each of these clusters. The identified cell populations include a majority of neuronal cells (83.4% of total sequenced cells) alongside a smaller fraction of non-neuronal cells (16.6%). Specifically, the neuronal clusters comprise Mki67^+^ intermediate neural progenitors (IPCs), immature Prox1^+^ Calb1^low^ and mature Prox1^+^Calb1^high^ Dentate Gyrus granule cells (imGCs and mGCs), Fibcd1^+^/Htr2c^+^/Prss35^+^ Cornu Ammonis (CA) neurons, Gad1^+^ inhibitory (GABA) neurons, and Trp73^+^Reln^+^ Cajal-Retzius (CR) cells. The non-neuronal populations include Aldh1l1^+^ astrocytes, Mbp^+^ oligodendrocytes, Pdgfra^+^ oligodendrocyte progenitor cells (OPCs), vascular Flt1^+^ endothelial cells, Col3a1^+^Ptgds^+^ fibroblast-like cells, immune Cd86^+^ microglia cells, and Foxj1^+^ ependymal cells (Fig. 1d, e, Suppl. Fig. 4). An interactive version of the data is available at https://hippo-seq.org/hi, featuring unintegrated datasets for each condition (sham, hypoxia-only, post-H-I) and an integrated dataset combining all conditions (shown in Fig. 1c). This platform allows for detailed exploration of hippocampal cell types under each condition and enables investigation of gene expression and co-expression patterns for user-defined genes and gene sets.

### Selective vulnerability of neonatal hippocampal neurons to hypoxia–ischemia

**Fig. 2:**
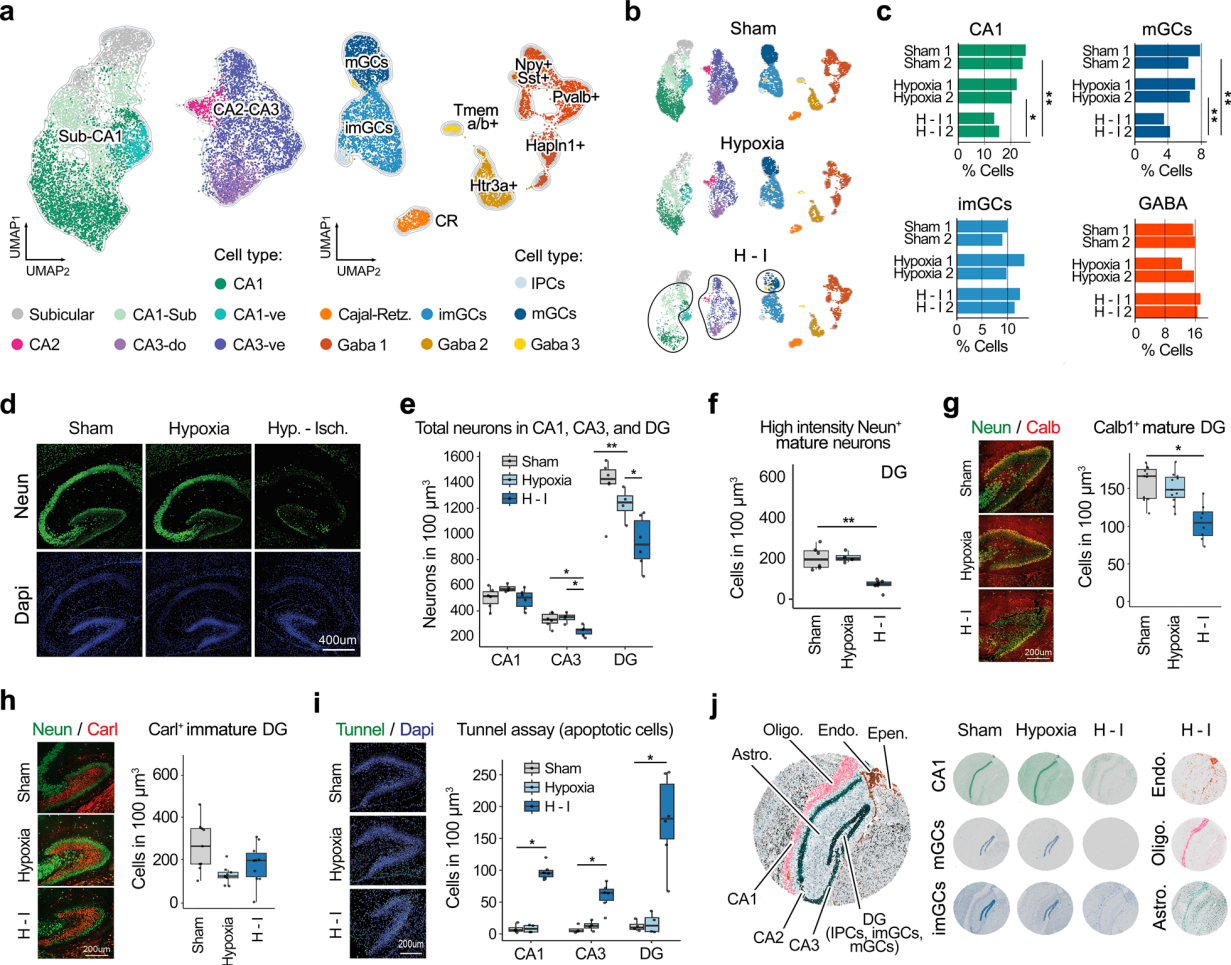
Early neuronal responses in the neonatal hippocampus to hypoxia and hypoxia–ischemia. **a** UMAP visualization of reclustered scRNA-seq data highlighting neuronal subtypes in the hippocampus including granule cells, Subiculum, CA1, CA2, CA3, Cajal-Retzius cells, and GABAergic neurons. **b** UMAP plots of neuronal subtypes visualized under three distinct conditions: sham (control), hypoxia alone, and H-I. **c** Quantitative analysis shows a significant proportional reduction in mature neuronal populations, especially in CA1 neurons and mature dentate gyrus granule cells, with immature granule cells and GABAergic neurons showing resilience. Statistical significance was determined using permutation tests with alternative hypothesis that values in hypoxia or hypoxia–ischemia groups are less than in sham group (one-sided test), *p = 0.05, **p < 0.05. **d** Representative images show NeuN + cells (green) and Dapi + nuclei (blue) in the hippocampus of P8 mice under three conditions. **e** Quantification of NeuN + cells per 100 um^3^ tissue volume across hippocampal subregions. Data are presented as mean ± SEM (n = 4–6 brain sections of 3 animals per condition), *p < 0.05, **p < 0.01 (two-sided Wilcoxon test). **f** Quantification of high-intensity NeuN + mature neurons per 100 um^3^ DG tissue volume. Data are presented as mean ± SEM (n = 4–6 brain sections of 3 animals per condition), **p < 0.01 (two-sided Wilcoxon test). **g** Representative images show NeuN + (green) and Calb + (red) mature granular cells (mGCs) in DG of P8 mice under each condition. Quantification of Calb + mGCs per 100 um^3^ DG tissue volume is presented alongside. Data are presented as mean ± SEM (n = 8–11 brain sections of 3–5 animals per condition), *p < 0.05. **h** Representative images show NeuN + (green) and Calr + (red) immature granular cells (imGCs) under sham, hypoxia-only, and H-I. Quantification of Calr + imGCs per 100 um^3^ DG tissue volume is presented alongside. Data are shown as mean ± SEM (n = 9–10 brain sections of 2–5 animals per condition). **i** Representative images show increased apoptotic cells (green) detected by the TUNEL assay in the mature DG granular cell layer after hypoxia–ischemia. Quantification of apoptotic cells 100 um^3^ tissue volume across hippocampal subregions is presented alongside. Data are presented as mean ± SEM (n = 4–6 brain sections of 3 animals per condition), *p < 0.05 (two-sided Wilcoxon test). **j** Alignment of distinct conditions: sham (control), hypoxia-only, and H-I with Slide-seq spatial transcriptomics data

To gain insights into the neuronal response to hypoxia-only and hypoxia–ischemia in the neonatal hippocampus, we performed a focused re-clustering of neuronal populations identified by our machine learning model. Subtypes of neurons were isolated and analyzed, including those from the subiculum, CA1, CA2, and CA3 areas, immature and mature granule cells (imGC and mGC) in the dentate gyrus (DG), Cajal-Retzius cells, and various GABAergic neurons characterized by specific markers (Htr3a^+^, Pvalb^+^, Hapln1^+^, Npy^+^Sst^+^, Tmem a/b^+^) (Fig. 2a). Post-hypoxia–ischemia, we observed a significant proportional cell type reduction in mature neurons, particularly in the CA and DG regions, with CA1 cells decreasing by 65.88%, mGCs by 54.31%, CA3 cells by 26.41%, and pro-subiculum neurons by 15.55% (Fig. 2b, c, Suppl. Fig. 5), consistent with prior findings of CA1's highest vulnerability to ischemia [35, 39, 59]. In contrast, imGCs, GABAergic neurons, and Cajal-Retzius cells remained statistically unaffected, indicating notable resilience. The reduction of hippocampal CA1/CA3 neurons and DG-mGCs was further validated by immunohistochemistry (Fig. 2d-i). The neuronal marker NeuN was used to detect both mature and immature hippocampal neurons, based on its expression levels reflected by strong and weak fluorescent intensity [9, 36] (Suppl. Fig. 6). A marked decrease in NeuN fluorescent intensity was observed following hypoxia–ischemia (Fig. 2d), along with a significant reduction in NeuN^+^ cells in the CA3 and DG (Fig. 2e, Suppl. Fig. 6c). Notably, the number of strongly NeuN-expressing CA1/CA3 neurons and mGCs was significantly reduced post-H-I (Fig. 2f, Suppl. Fig. 6c). This reduction in mGCs was further validated by the mGC-specific marker Calbindin (Calb1^+^) (Fig. 2g), while no significant decrease in Calretinin-positive (Calr^+^) imGCs was detected (Fig. 2h). Additionally, the TUNEL assay revealed a significant increase of apoptotic cells across all hippocampal subregions, particularly in the DG-mGC layer (Fig. 2i). Taken together, these results underscore the vulnerability of mature hippocampal neurons to acute stress following hypoxic-ischemic brain injury.

To better understand the spatial and molecular context of these changes, we aligned our scRNA-seq data with Slide-seq spatial transcriptomics of the mouse hippocampus [67]. This alignment revealed that hypoxia alone did not significantly alter gene expression profiles, allowing consistent matching with Slide-seq data (Fig. 2j). In contrast, post-hypoxia–ischemia, neurons in hippocampal subregions became poorly aligned, while non-neuronal cells such as endothelial cells, microglia, and astrocytes remained mostly unaffected. Notably, even the relatively resilient immature granule cell (imGC) population showed similar misalignment, indicating that transcriptional profiles and RNA integrity were compromised post-injury in various neuronal populations, reflecting complex stress-induced alterations.

### Acute transcriptional signatures of neuronal resilience and vulnerability to hypoxia–ischemia in the neonatal hippocampus

**Fig. 3:**
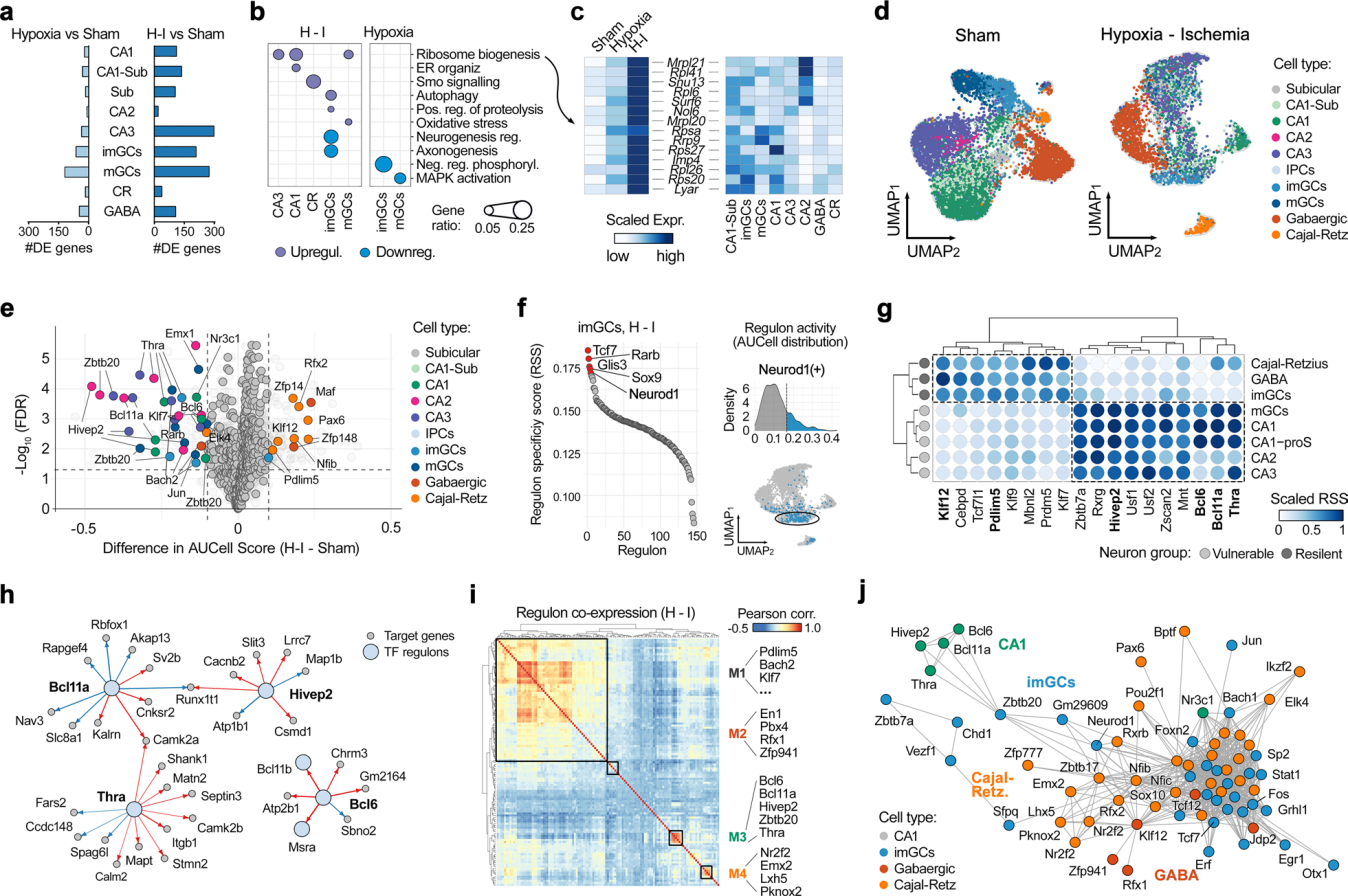
Cell type-specific transcriptional responses and key transcription factors underlying neuronal vulnerability and resilience post-H-I. **a** Differential expression analysis of neuronal subtypes under the post-hypoxia and hypoxia–ischemia conditions compared to sham (FDR < 0.1, |log_2_FC|> 1). **b** Gene set enrichment analysis (GSEA) of neuronal subtypes post-hypoxia–ischemia. **c** Scaled average expression of ribosome biogenesis-related genes across neuronal subtypes under sham, hypoxia-only, and H-I conditions (left), and their expression across various neuronal cell types following H-I (right). **d** UMAP plots of cells re-clustered based on regulon activity profiles (AUCell scores) for sham and post-H-I conditions. **e** Volcano plot displaying differential regulon activity between H-I and sham conditions (FDR < 0.05, |AUCell difference|> 0.1). **f** Left panel: Regulons specific to imGCs ranked by regulon specificity score (RSS), see methods. Higher RSS indicates greater specificity for imGCs compared to other cell types. Top 5 regulons are shown in red. Right panel: AUCell score histogram for the Neurod1 regulon in imGCs and UMAP plot under H-I conditions, highlighting cells with high (blue) and low (grey) regulon activity (right), demonstrating strong specificity to imGCs. **g** Regulons specific to vulnerable or resilient neurons post-hypoxia–ischemia. Regulons with significantly altered activity (as shown in panel e) and high specificity to vulnerable or resilient groups are highlighted in bold. **h** Gene regulatory network (GRN) depicting regulons Hivep2, Bcl11a, Bcl6, and Thra and their top downstream target genes with the most altered regulatory importance after hypoxia–ischemia. **i** Pairwise correlation matrix of transcription factors in neuronal subtypes, revealing distinct co-expression modules: M1 (common across cell types), M2 (GABAergic neurons), M3 (CA1 pyramidal neurons), and M4 (Cajal-Retzius cells). **j** Interaction network of transcription factors within each co-expression module. Nodes represent TFs, colored by the cell type with the highest AUCell score. Edges represent correlations between TFs (using a Pearson correlation threshold of *ρ* > 0.45)

To further dissect transcriptional changes in distinct neuronal cell clusters, we performed differential gene expression (DE) analysis under both post-hypoxia (hypoxia-only vs sham) and post-hypoxia–ischemia (H-I vs sham) conditions. A significant increase in differentially expressed genes (DEGs) was observed across all neuronal subtypes following hypoxia–ischemia, with the majority being upregulated (Fig. 3a, Suppl. File 1), highlighting a pronounced ischemia-induced transcriptional shift.

Gene set enrichment analysis revealed distinct molecular adaptations in affected neuronal populations (Fig. 3b). Specifically, upregulated ribosomal biogenesis was identified in neuronal populations exhibiting increased apoptosis (CA1-3 and mGCs) following hypoxia–ischemia (Fig. 3b). Genes related to ribosome biogenesis (e.g., Nol6, Imp4, Surf6, Rrp9, Rpl6, Rpl26, Rpl41, Rps20) were predominantly upregulated in these neurons (Fig. 3c). This response was absent in GABAergic neurons and Cajal-Retzius cells, as well as non-neuronal cells (Suppl. Fig. 7a), suggesting that affected neurons initiate an acute transcriptional response prioritizing protein synthesis for repair, despite the energy crisis induced by hypoxia–ischemia. Immature granule cells (imGCs) showed upregulation of pathways related to autophagy (e.g., Atg12, Becn1, Gabarapl2, Sesn2) and proteolysis (e.g., Furin, Psmc3, Ctsl). Similar patterns, though not statistically significant across all populations, were observed in all neuronal types (Suppl. Fig. 7b), implying conserved stress-response mechanisms in neurons following hypoxia–ischemia [10, 40]. Additionally, imGCs exhibit specific downregulation of pathways associated with neurogenesis (e.g., Prox1, Ntf3, Sema3c) and axon extension (e.g., Sema5a, Sema3c, Myo5b), suggesting that imGCs prioritize survival over growth by conserving resources and stabilizing existing neuronal structures rather than promoting new development. Interestingly, Cajal-Retzius cells show upregulated genes (Arl13b, Traf3ip1, Hspg2, Dcdc2a) associated with Smoothened signaling, a key component of the neuroprotective Hedgehog pathway, known for its role in promoting cell survival and neuroprotection [42] (Fig. 3b, Suppl. File 1). Taken together, these transcriptional changes underlie the observed increased vulnerability of excitatory neurons in CA1-3 and mGCs in DG post-H-I, while also supporting the greater resilience observed in GABAergic neurons, Cajal-Retzius cells, and imGCs.

Next, we utilized SCENIC (Single-Cell rEgulatory Network Inference and Clustering) analysis to reconstruct cell type-specific gene regulatory networks (GRNs) and identify key transcription factors (TFs) underlying neuronal vulnerability and resilience after H-I. Cells were reclustered based on their regulon activity profiles using AUCell (see methods), with neuronal cell types remaining largely separated post-ischemia, suggesting preserved core regulatory identities despite stress-induced transcriptional changes (Fig. 3d). Differential regulon activity analysis between H-I and sham conditions revealed TFs with significantly altered regulatory influence, including downregulated Hivep2, Bcl6, Bcl11a, Thra, Zbtb20, Bach2, Klf7, Emx1, Nr3c1, Rarb, Jun, and Elk4, and upregulated Pdlim5, Zfp148, Zfp14, Klf12, Pax6, Maf, Nfib (Fig. 3e). To characterize cell-type-specific regulatory roles of these TFs, we performed regulon specificity analysis, identifying key regulons for each cell type (Suppl. File 2), such as Neurod1 for imGCs (Fig. 3f). Strikingly, common regulons were identified in vulnerable and resilient hippocampal cell populations post-H-I (Fig. 3g), highlighting the distinct molecular responses that underly cell-type-specific reactions to hypoxia–ischemia.

Integration of differential regulon activity and regulon specificity analyses revealed that certain regulons, including Hivep2, Bcl6, Bcl11a, and Thra, exhibited both decreased activity (Fig. 3e) and increased specificity (Fig. 3g) in vulnerable neurons post-ischemia, suggesting their critical role in neuronal susceptibility to damage. Conversely, Klf12 and Pdlim5 regulons demonstrated upregulated activity and higher specificity in resilient neurons, indicating their potential importance in neuronal survival under ischemic conditions (Fig. 3g, marked in bold). Delving deeper into the transcription factors linked to neuronal vulnerability, we found that Hivep2, Bcl11a, Bcl6, and Thra significantly impacted genes involved in calcium homeostasis and signaling (Fig. 3h). Hivep2 showed increased regulatory importance for Cacnb2 (voltage-gated calcium channel subunit) and decreased influence on Atp1b1 (Na⁺/K⁺-ATPase subunit) as well as on Prkcg, Cpne6, and Grm7 (calcium-dependent neuronal signaling and function). Bcl11a exhibited increased regulatory importance for Camk2a (calcium/calmodulin-dependent protein kinase II alpha) and decreased importance for Slc8a1 (sodium/calcium exchanger). Bcl6 demonstrated increased regulatory influence on Atp2b1 (plasma membrane calcium ATPase 1), Chrm3 (muscarinic acetylcholine receptor M3), and Grin2a (NMDA receptor subunit 2 A). Conversely, Thra exhibited decreased regulatory influence on calcium-related genes, including Camk2a, Camk2b, and Calm2 (calmodulin 2). The altered regulatory influence of key transcription factors on calcium-related genes underscores the critical role of calcium dysregulation in promoting neuronal death, aligning with the well-established mechanisms of calcium-dependent excitotoxicity in neurons. In addition, Hivep2 decreased regulation of Atp1b1; Bcl11a altered Kalrn and Rbfox1; Bcl6 modified Bcl11b, Negr1, and Lsamp; and Thra reduced influence on Itgb1, Matn2, Dync1i2, Mapt, Stmn2, Sptbn2, and Shank1. These changes in genes related to ion homeostasis, synaptic function, neuronal differentiation, adhesion, and cytoskeletal organization may contribute to neuronal vulnerability post-injury.

To further elucidate the regulatory landscape, we performed regulon co-expression analysis by calculating pairwise correlations of transcription factors (TFs) in neuronal cell types and identified four distinct modules, specific to different neuronal cell types (Fig. 3i). A correlation matrix was used to visualize interactions between TFs within each module, with each TF colored according to the cell type with the highest AUCell score (Fig. 3j). This analysis revealed that the transcription factors Hivep2, Bcl6, Bcl11a, and Thra clustered together, further suggesting a coordinated regulatory program with potential synergistic action of these TFs in regulating calcium-related genes in the vulnerable neuronal cell types.

### Rapid glial activation in the neonatal hippocampus following hypoxia–ischemia

**Fig. 4:**
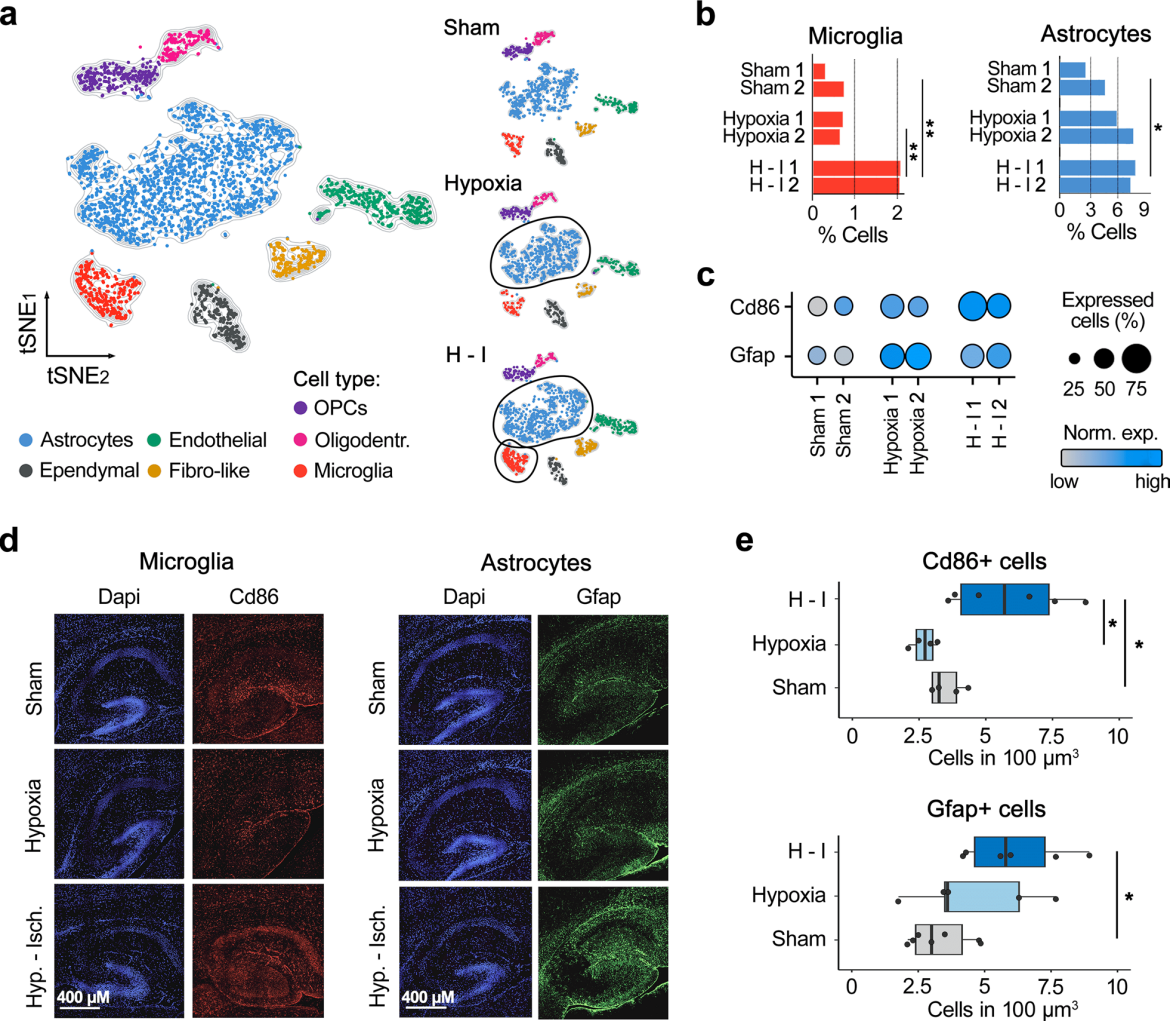
Early non-neuronal responses in the neonatal hippocampus to hypoxia and hypoxia–ischemia. **a** UMAP visualization of reclustered scRNA-seq data highlighting non-neuronal cell subtypes in the hippocampus. **b** Proportional changes in the populations of activated microglia and astrocytes across sham (control), hypoxia-only, and hypoxia–ischemia conditions. **c** Expression levels of Cd86 in microglia and Gfap in astrocytes under different conditions. **d**. Representative immunohistochemistry images of hippocampal tissue stained for Cd86 (microglia) and Gfap (astrocytes) in control, post-hypoxia, and post-hypoxia–ischemia conditions. **e** Quantification of immunohistochemistry staining intensity for Cd86 and Gfap. Data are presented as mean ± SEM (n = 4–7 brain sections of 2–3 animals per condition), *p < 0.05 (two-sided Wilcoxon test)

Next, we expanded our analysis to non-neuronal cell populations by reclustering and annotating them using our machine learning-based approach, identifying seven distinct cell types: astrocytes, microglia, oligodendrocytes, oligodendrocyte precursor cells (OPCs), endothelial cells, fibroblast-like cells, and ependymal cells (Fig. 4a). Remarkably, just three hours after the H-I event, we observed a significant proportional increase in activated microglia and astrocytes (Fig. 4b). This rapid increase suggests cellular activation rather than proliferation, as evidenced by elevated expression of Cd86 in microglia and Gfap in astrocytes—both markers associated with reactive gliosis [17, 30]—implying an immediate neuroinflammatory response following H-I injury (Fig. 4c). Interestingly, when examining the effects of hypoxia alone, without concurrent ischemia, only astrocytes exhibited increased activation, with no significant changes observed in microglia (Fig. 4b,c). The upregulation of activated microglia (Cd86 +) and astrocytes (Gfap +) post-hypoxia–ischemia was confirmed by immunohistochemistry (Fig. 4d, e). Statistical analysis using a two-sided Wilcoxon test supported these findings, reinforcing the notion of a rapid glial response to H-I injury.

### Multifaceted early transcriptional responses in hippocampal non-neuronal cells to hypoxia–ischemia

**Fig. 5:**
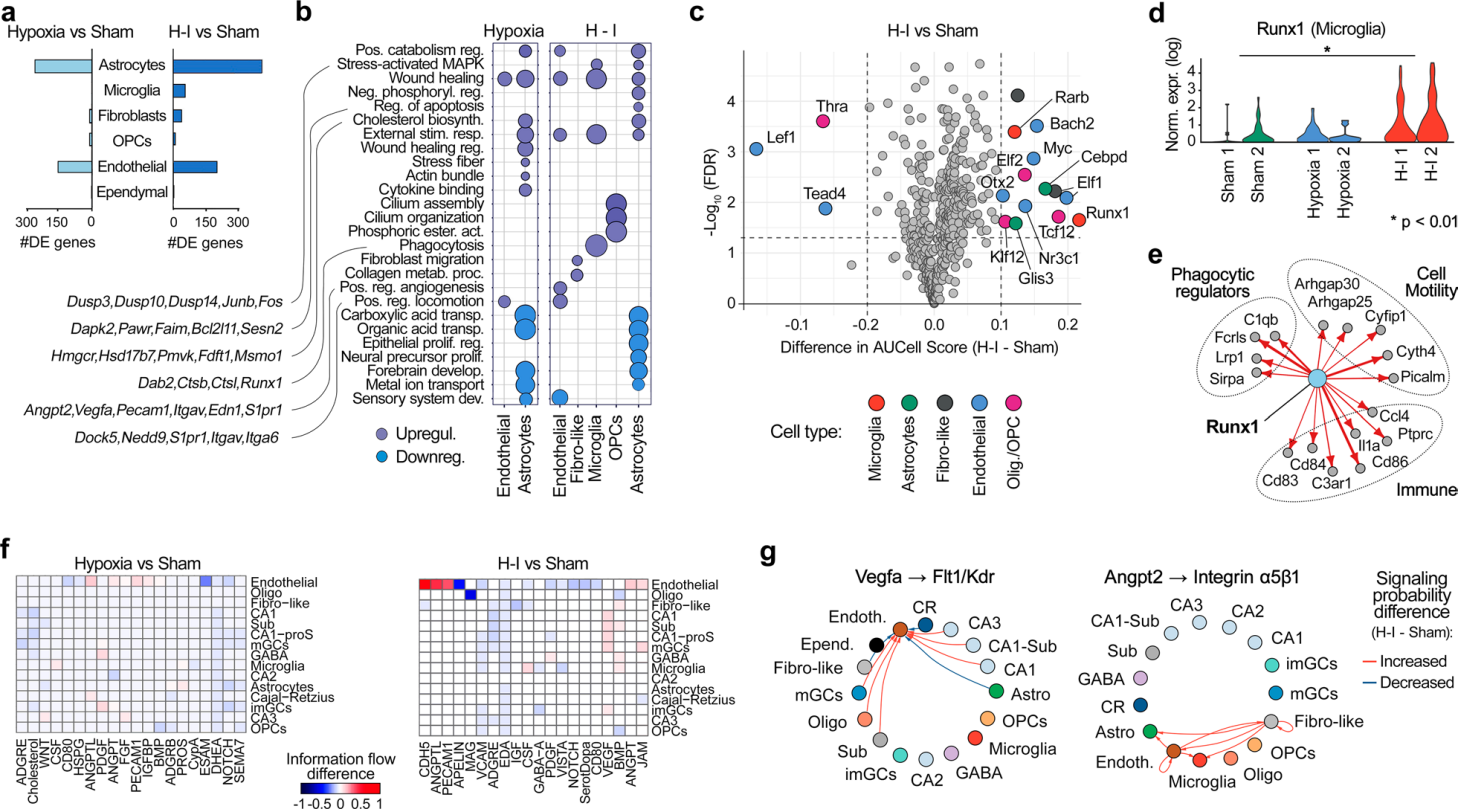
Early transcriptional responses in non-neuronal populations. **a** Differential expression analysis of non-neuronal cells under hypoxia-only versus sham and H-I versus sham conditions. Bars represent the number of DE genes (false discovery rate [FDR] < 0.1, |log_2_fold change|> 1). **b** Gene set enrichment analysis (GSEA) of non-neuronal cell populations under hypoxia-only and H-I conditions. **c** Volcano plot illustrating differential regulon activity between H-I and sham conditions. Significantly up- and down-regulated regulons are labeled (FDR < 0.05, |AUCell difference|> 0.1). **d** Violin plots depicting Runx1 expression levels in microglia under sham, hypoxia-only, and H-I conditions. **e** Gene regulatory network (GRN) of Runx1 and its upregulated downstream targets in microglia. **f** Heatmaps illustrating differential intercellular signaling patterns quantified as changes in information flow between hypoxia-only vs sham (left) and H-I vs sham (right). Information flow, derived from CellChat analysis, represents the aggregate strength and probability of ligand-receptor interactions between cell populations (see methods). The color scale indicates the increased (red) and decreased (blue) changes in information flow. **g** Circular plots depicting signaling probability differences for VEGFA pathway via Flt1/Kdr receptors (left) and ANGPT2 pathway via integrin α5β1 receptors (right). Line color indicates the increase (red) or decrease (blue) in signaling probability

Similarly, we performed differential gene expression analysis in non-neuronal cell populations, comparing hypoxia-only versus sham and hypoxia–ischemia versus sham conditions to identify early transcriptional changes in these cell types. Astrocytes exhibited hundreds of DEGs in both hypoxia-only and hypoxia–ischemia conditions, indicating a transcriptional shift in astrocytes in response to both stressors (Fig. 5a). Gene set enrichment analysis revealed that astrocytes, activated under both hypoxia-only and H-I, exhibited a multifaceted response (Fig. 5b). Under both conditions, the cholesterol biosynthesis pathway was upregulated, indicated by increased expression of genes such as Hmgcr, Hsd17b7, Pmvk, Fdft1, Msmo1, and Lbr, which are essential for membrane repair, synaptic maintenance, and neuroprotection. Additionally, genes involved in positive regulation of catabolic processes (Lpl), wound healing (Cd44, Pdgfa, Hbegf, Tgfb1), and response to external stimuli (Atf3, Map3k14, Nrros) were upregulated, suggesting that astrocytes enhance metabolic activity, facilitate tissue repair, and adapt to stress signals following hypoxic insult. Following ischemia, astrocytes uniquely upregulated genes involved in the regulation of apoptotic processes, including Dapk2, Pawr, Faim, Bcl2l11, and Sesn2, indicating a response to increased cellular damage by modulating apoptosis to balance survival and programmed cell death. Genes associated with the stress-activated MAPK pathway—such as Dusp3, Dusp10, Dusp14, Junb, and Fos—were also specifically upregulated after ischemia, which may enable astrocytes to manage heightened stress signals, regulate inflammatory responses, and initiate repair mechanisms. Furthermore, upregulation of genes related to cytoskeletal remodeling and cell migration, including Rnd1, Amotl2, Itga1, Thbs1, and Ror2, suggests that astrocytes begin preparing for migration to H-I injury sites even at this early time point.

The significantly increased DEGs were also identified in microglia, fibroblasts, and endothelial cells following H-I, with the majority being upregulated (Fig. 5a, Suppl. File 3). Microglia responded specifically to H-I injury by upregulating genes related to phagocytosis (e.g., Dab2, Ctsb, Ctsl), response to external stimuli (Runx1, Tgfb1, Prkch), and wound healing (Tgfbi, Tgfb1). SCENIC analysis identified Runx1 as a key transcriptional regulator in the microglial response following H-I injury (Fig. 5c). Notably, Runx1 itself showed significant upregulation in both expression levels and the proportion of microglia expressing it (Fig. 5d). Runx1 exhibited increased regulatory activity on diverse downstream targets encompassing several crucial aspects of microglial function (Fig. 5e). These targets include genes involved in immune activation (e.g., Cd86, Ptprc/Cd45, C3ar1, Ccl4, Cd84, Cd83, Il1a), phagocytosis and debris clearance (Fcrls, Sirpa, Lrp1, C1qb), and cytoskeletal remodeling and migration (Cyth4, Picalm, Arhgap25, Arhgap30, Cyfip1). The extensive influence of Runx1 across these functional categories underscores its potential role as a master regulator orchestrating multiple aspects of the acute microglial response to H-I injury in the developing brain. Additionally, intercellular signaling analysis suggests increased autocrine signaling of microglia through Csf, especially post-H-I, while Csf1 regulatory activity was mostly increased by Runx1 (Fig. 5f).

DE and SCENIC analyses revealed a complex transcriptional response of endothelial cells post-ischemia. We observed transcriptional downregulation and decreased activity of the Tead4 and Lef1 TFs, involved in developmental gene regulation and cellular differentiation [14, 88] (Fig. 5c). Conversely, there was increased activity in the Otx2, Nr3c1, Myc, Bach2, and Elf1 regulons, and key angiogenic genes such as Angpt2, Vegfa, Pecam1, Itgav, Edn1, and S1pr1 were transcriptionally upregulated (Fig. 5b). These findings suggest that endothelial cells respond to ischemia by downregulating certain developmental transcription factors while simultaneously activating angiogenic pathways to promote vascular repair. Additionally, DE analysis identified upregulation of pathways related to wound healing and positive regulation of locomotion, with increased expression of genes such as Dock5, Nedd9, S1pr1, Itgav, and Itga6, suggesting that endothelial cells are initiating processes related to vascular remodeling and maintenance of blood–brain barrier integrity in response to hypoxic stress (Fig. 5b).

Fibroblast-like cells exhibited upregulation of genes associated with migration and collagen metabolism—processes important for vascular remodeling and wound healing after ischemia (Fig. 5b). At 3 h post-ischemia, increased expression of genes such as Fgf2, Itga5, Cd44, and Tns1 suggests initiation of signaling pathways related to migration, even though significant cell movement is unlikely to occur at this early stage. Similarly, upregulation of collagen metabolism genes—including Mmp19, Mmp28, Ctsl, Col5a3, Col6a1, and Hspg2—indicates early activation of extracellular matrix remodeling and synthesis. These findings suggest that fibroblast-like cells begin engaging in pathways associated with tissue repair soon after ischemic injury.

### Altered intercellular communication in the neonatal hippocampus following hypoxia–ischemia

The cell–cell communication analysis revealed substantial changes in the signaling information flow among neuronal and non-neuronal cells following hypoxia-only and hypoxia–ischemia (Fig. 5f). Endothelial cells exhibit the most pronounced alterations in their signaling pathways following H-I, particularly with an upregulation in endothelial autocrine signaling involving VE-cadherin (Cdh5) and PECAM1 (Cd31), and downregulation of the Apelin signaling pathway (Fig. 5f). The upregulation of VE-cadherin and PECAM1 signals is essential for endothelial cell junctions, controlling vascular stability and permeability. In contrast, the downregulation of Apelin signaling, which regulates angiogenesis and vascular tone, may reflect early endothelial adaptations to modulate angiogenesis and stabilize the vasculature in the injured brain. Additionally, endothelial cells displayed altered communication with astrocytes, microglia, and other non-neuronal cells through increased Angpt2 signaling via the integrin α5β1 receptor (Fig. 5g, right panel). Angiopoietin-2 (Angpt2) is involved in vascular remodeling and inflammation [69], suggesting that endothelial cells may engage in crosstalk with surrounding cells to coordinate vascular repair processes and modulate immune responses. These signaling shifts in endothelial cells and among intercellular communications highlight the pivotal role of endothelial cells in modulating the vascular response to hypoxia–ischemia, impacting neuroprotection, neuroinflammation, vascular remodeling, and recovery processes.

Strikingly, vulnerable neurons (CA1, CA3, mGCs) exhibited increased communication with endothelial cells via the vascular endothelial growth factor (VEGF) signaling pathway. Upregulation of Vegfa signaling and interactions with its receptors Flt1 (VEGFR-1) and Kdr (VEGFR-2) on endothelial cells may serve as early signals initiating angiogenesis (Fig. 5g, left panel). This finding complements the observed upregulation of angiogenesis-related genes in endothelial cells, suggesting a coordinated response between vulnerable neurons and endothelial cells to restore oxygen supply following ischemic insult. Conversely, resilient neurons such as imGC and GABAergic neurons did not show significant changes in cell communication pathways. This suggests that their resilience is likely maintained through the enhancement of existing signaling mechanisms and autonomous protective strategies, rather than through the activation of new intercellular communication routes.

## Discussion

Neonatal hypoxic-ischemic brain injury is a leading cause of neurodevelopmental disabilities and is responsible for 23% of infant mortality [19, 26, 74]. The hippocampus is particularly vulnerable to H-I due to its high metabolic demands and critical role in cognitive functions [15, 19, 47, 90]. In this study, we provide novel insights into the immediate cellular and transcriptional responses of the neonatal hippocampus following H-I injury, using single-nucleus RNA sequencing (snRNA-seq) combined with a machine learning-based cell annotation framework. By examining the acute phase, just three hours post-ischemic injury, we uncovered cell-type-specific vulnerability patterns, adaptive stress responses, and key transcriptional regulators that may influence long-term neurological outcomes. By constructing a comprehensive reference atlas and training our model on it, we achieved high specificity in cell type identification, enabling the detection of subtle changes in cell states and regulatory networks that might be overlooked using conventional methods. This machine learning-based framework enhances the resolution and reliability of our findings and sets a new standard for interrogating cellular heterogeneity and dynamics in complex tissues under various physiological and pathological conditions. Our hippocampal reference atlas was constructed using datasets from mice aged 1.5 months and older due to the limited availability of high-quality annotated data from the neonatal hippocampus. Future work could incorporate neonatal transcriptomic data as it becomes available to further refine the atlas and enhance classification accuracy for developmental studies.

A principal finding of our study is the acute susceptibility of mature hippocampal neurons to H-I injury. We observed a significant reduction in mature CA1 and CA3 pyramidal neurons and mature dentate gyrus granule cells (mGCs) corroborated by immunohistochemical analyses using neuronal marker NeuN and mGC-specific marker Calbindin. Consistently, increased apoptosis was detected in these populations. The rapid loss of these neurons underscores a critical therapeutic window in which interventions might prevent irreversible damage and preserve neurological function. Notably, upregulated ribosome biogenesis was detected in vulnerable neurons within CA1-3 and GC regions, possibly as an attempt to restore damaged cellular components. However, this energy-intensive process [31] may exacerbate neuronal stress under H-I conditions due to limited energy resources during insult [27, 84]. Dysregulated ribosome biogenesis was shown to contribute to disease progression and worsen ischemic stroke outcomes by driving inflammation and neuronal stress [31, 76], making it a promising therapeutic target to mitigate cellular damage and improve recovery.

In contrast, certain neuronal subtypes exhibited remarkable resilience to H-I. Immature granule cells (imGCs), GABAergic interneurons, and Cajal-Retzius (CR) cells maintained their proportions and displayed distinct transcriptional profiles suggestive of adaptive stress responses. imGCs upregulated autophagy and proteolysis pathways while downregulating genes associated with neurogenesis and axon extension. This suggests a strategic shift towards energy conservation and survival under stress conditions, potentially attributed to their developmental stage, as immature neurons often possess greater plasticity and adaptability to stress [54]. However, while proteolysis supports cellular maintenance in imGCs, modulating proteolysis—to balance protein cleanup without promoting excessive breakdown—is essential, as studies show that inhibition of proteolysis can reduce neuronal death after H-I [40]. These findings highlight the potential of targeting imGCs, with carefully modulated proteolytic pathways, to promote endogenous repair and neurogenesis following H-I injury. CR cells upregulated genes associated with the Hedgehog signaling pathway, including components of Smoothened signaling, known for their roles in neuroprotection, cell survival, and differentiation [42]. Activation of this pathway may enable CR cells to withstand hypoxic-ischemic stress, contributing to their resilience.

Through differential expression and gene regulatory network analyses, we identified key transcription factors (TFs) associated with neuronal vulnerability and resilience. In vulnerable neurons, we observed downregulation of TFs such as Hivep2, Bcl11a, Bcl6, and Thra, which are crucial for neuronal development, synaptic function, and calcium homeostasis [5, 13, 66, 79]. Altered regulation of calcium-related genes by these TFs may contribute to excitotoxic neuronal death, a well-established mechanism in ischemic injury [51, 62]. For instance, dysregulation of Hivep2 and Bcl11a has been implicated in neurodevelopmental disorders and synaptic dysfunction [13, 66], and our findings suggest their roles extend to mediating neuronal vulnerability in H-I injury. Notably, Hivep2 and Bcl6 are critical transcription factors in the development of adult-born neurons [52], suggesting their downregulation may impact both initial injury responses and subsequent regenerative processes. These findings underscore the potential of targeting these TFs to stabilize calcium homeostasis and mitigate excitotoxicity, providing a potential therapeutic approach to ischemic injury.

We observed a rapid activation of astrocytes and microglia in response to H-I, evidenced by increased expression of Gfap and Cd86, respectively. Astrocytes upregulated cholesterol biosynthesis pathways, essential for membrane repair and synaptic stability [50], and genes involved in wound healing and stress responses. The upregulation of genes regulating apoptosis and the MAPK pathway indicates that astrocytes are actively modulating cell death and inflammatory signaling following injury. However, prolonged astrocyte activation shows both beneficial and detrimental effects on neural plasticity and functional recovery [49], suggesting that therapeutic strategies should modulate astrocyte function rather than broadly suppress or enhance their responses.

Microglia exhibited upregulation of genes associated with phagocytosis, immune activation, and wound healing, with Runx1 (AML1) emerging as a key TF orchestrating these responses. Known for regulating microglial proliferation and differentiation during development, Runx1 exhibited increased activity on downstream targets involved in immune activation, debris clearance, cytoskeletal remodeling, and migration [33, 44]. This underscores its role as a master regulator of the acute microglial response to hypoxia–ischemia injury in the developing brain. Enhanced autocrine and paracrine signaling of microglia via CSF1 and VEGFA pathways underscores the dynamic interplay between microglia, neurons, and endothelial cells in coordinating early immune responses and initiating repair mechanisms. Modulating Runx1 activity could help balance beneficial and detrimental microglial functions in neonatal brain injury.

Endothelial cells displayed a complex transcriptional response post-H-I, characterized by downregulation of developmental TFs (Tead4 and Lef1), increased activity of angiogenic TFs (Myc and Elf1), and upregulation of pro-angiogenic TFs (Vegfa, Angpt2, and Pecam1). This suggests an immediate attempt to initiate vascular repair and restore oxygen supply, shifting from the developmental program. The increased interaction between vulnerable neurons and endothelial cells via VEGFA signaling highlights the significance of neurovascular communication in responding to ischemic injury. Altered signaling involving Angpt2 and integrins indicates endothelial involvement in modulating vascular permeability and inflammation [69]. These adaptations may facilitate revascularization and support the restoration of cerebral blood flow.

Our findings underscore the critical importance of the early post-injury period in shaping outcomes following neonatal H-I. The rapid loss of mature neurons suggests that timely interventions aimed at preventing excitotoxicity and stabilizing calcium homeostasis could be particularly effective. Targeting the identified TFs associated with neuronal survival and death pathways offers potential strategies for therapeutic modulation. Enhancing the activity of protective TFs or signaling pathways in vulnerable neurons, or leveraging resilience mechanisms in GABAergic neurons and CR cells, could mitigate neuronal loss. Moreover, the activation of astrocytes and microglia presents both challenges and opportunities. While glial responses are essential for debris clearance and initiating repair, excessive activation can lead to inflammation and secondary injury [61, 83]. Therapeutic approaches that modulate glial activation to enhance protective functions while minimizing detrimental effects could improve recovery outcomes. Supporting endothelial cell function and promoting angiogenesis might also facilitate the restoration of cerebral blood flow, reduce tissue damage, and accelerate recovery processes.

This study provides a comprehensive assessment of immediate cellular and molecular responses within the therapeutic window post-hypoxia–ischemia, revealing cell-type-specific vulnerability and neuroprotective mechanisms. It should be noted that our analysis utilized two mice per condition (balanced for sex), which represents a limitation in fully capturing biological variability. While this design allowed identification of robust transcriptional patterns across cell types, the small sample size may have limited our ability to detect more subtle cell-type-specific changes. Future studies with larger cohorts, including both sexes in different conditions, would substantially enhance our understanding of the heterogeneity in cellular responses to hypoxic-ischemic injury. Future work will also incorporate multiple time points post-H-I to capture the temporal progression of cellular and transcriptional changes. Longitudinal studies are necessary to understand the dynamics of these responses and identify critical windows for intervention. An important clinical extension would be investigating how therapeutic hypothermia—the current standard of care for neonatal H-I [92]—modulates these early cellular responses, as this would inform the development of adjuvant therapies. Additionally, examining whether similar mechanisms exist in milder H-I injuries could address an important clinical gap. Integrating multi-omics approaches, such as metabolomics, proteomics, post-translational modifications (PTMs), epitranscriptomics, and epigenetics, would offer a more comprehensive understanding of the underlying regulatory mechanisms.

## Conclusion

Our comprehensive single-cell analysis elucidates the immediate cellular and transcriptional adaptations in the neonatal hippocampus following hypoxia–ischemia. The selective vulnerability of mature neurons, contrasted with the resilience of immature granule cells, GABAergic neurons, and Cajal-Retzius cells, underscores the complexity of neuronal responses to injury. The rapid activation of glial and endothelial cells further highlights the multifaceted nature of the early post-injury environment. By identifying key transcriptional regulators and signaling pathways, our study provides a foundation for developing targeted interventions aimed at mitigating damage and promoting repair in neonatal hypoxic-ischemic encephalopathy. The developed publicly accessible data and analytical tools will serve as valuable resources for the neuroscience community to explore hippocampal cell heterogeneity and pathophysiology.

## Supplementary Information

Additional file 1.

Additional file 2.

Additional file 3.

Additional file 4.

## Data Availability

SnRNA-seq data produced in this study are available at https://hippo-seq.org/download and interactive versions of datasets are available at https://hippo-seq.org/hca and https://hippo-seq.org/hi.

## Abbreviations

H-I: Hypoxic-ischemic
snRNA-seq: Single-nucleus RNA sequencing
P8: Postnatal day 8
DOB: Date of birth
HPC: Hippocampus
CA: Cornu ammonis
DG: Dentate gyrus
HCA: Hippocampal cell atlas
IPCs: Intermediate neural progenitors
CR cells: Cajal-Retzius cells
OPCs: Oligodendrocyte progenitor cells
mGC: Mature granule cells
imGC: Immature granule cells
DE: Differential gene expression
DEGs: Differentially expressed genes
SCENIC: Single-cell regulatory network inference and clustering
GRNs: Gene regulatory networks
TFs: Transcription factors
GSEA: Gene set enrichment analysis
