# Supplementary material for: Early transcriptional responses reveal cell type-specific vulnerability and neuroprotective mechanisms in the neonatal ischemic hippocampus
Source: Acta Neuropathol Commun. 2025 Jul 5;13:147. doi: 10.1186/s40478-025-02062-4 (PMC12228329; doi:10.1186/s40478-025-02062-4)
Supplement: Supplementary file 1 — Additional file 1. [file 40478_2025_2062_MOESM1_ESM.pdf]

Supplementary figures

Early transcriptional responses reveal cell type-specific vulnerability and neuroprotective mechanisms in the neonatal ischemic hippocampus

Aleksandr Ianevski[1]*, Maria Camara Quilez[1], Wei Wang[1], Rajikala Suganthan[2,3], Gunn Hildrestrand[2,3], Jonas Viken Grini[1], Dagny Sanden Døskeland[1], Jing Ye[1]*, Magnar Bjørås[1,2,3]*

[1]Department of Clinical and Molecular Medicine (IKOM), Norwegian University of Science and Technology (NTNU), 7491, Trondheim, Norway.
[2] Department of Microbiology, Oslo University Hospital, University of Oslo, Oslo, 0424, Norway
[3]Centre for Embryology and Healthy Development, University of Oslo, Oslo, 0373, Norway

*Correspondence: Aleksandr Ianevski aleksandr.ianevski@ntnu.no
Jing Ye jing.ye@ntnu.no
Magnar Bjørås magnar.bjoras@ntnu.no

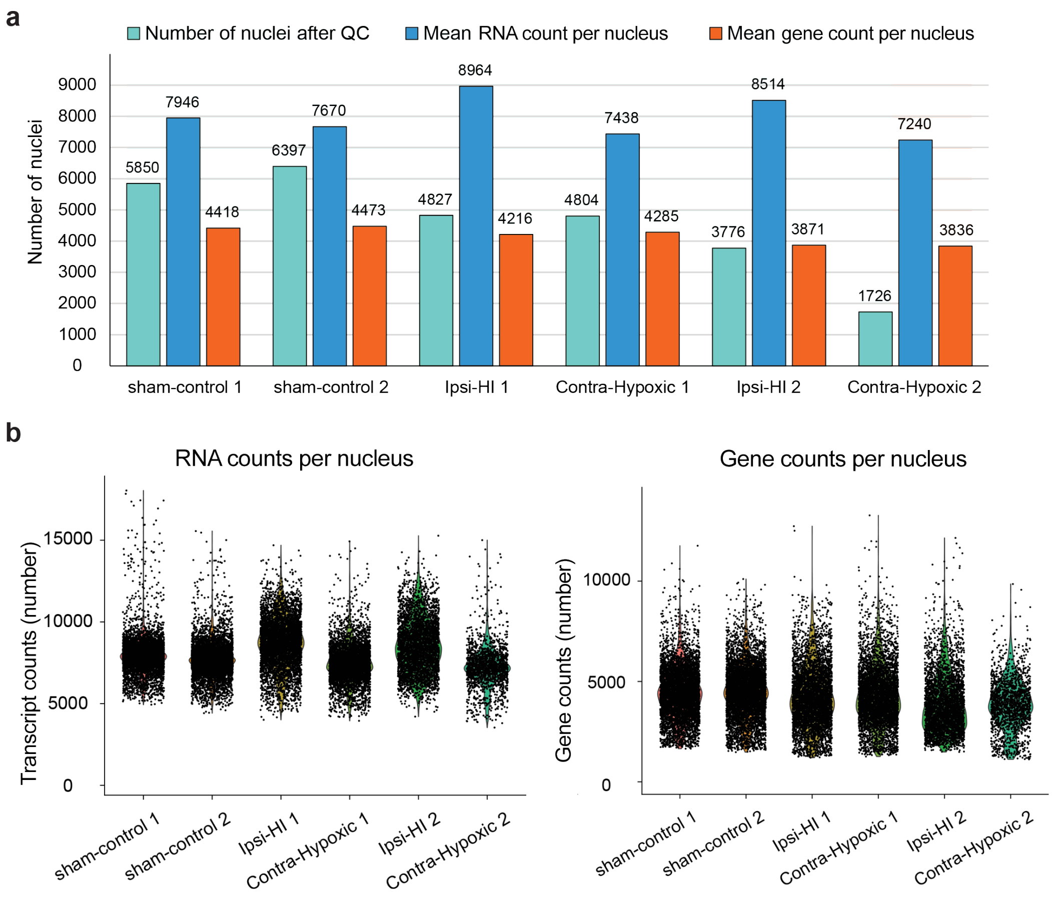

Supplementary Figure 1. Quality control metrics of hippocampal scRNA-seq samples from perinatal mice subjected to different experimental conditions: sham treatment, 45-minute hypoxia, and 3-hour post-hypoxia-ischemia snRNA-seq data. (a) Bar plot showing the number of nuclei passing quality control (light green), mean RNA counts per nucleus (blue), and mean gene counts per nucleus (orange) for each sample. (b) Distribution of transcript counts per nucleus (left) and gene counts per nucleus (right) across all nuclei in each condition and replicate. Each dot represents an individual nucleus.

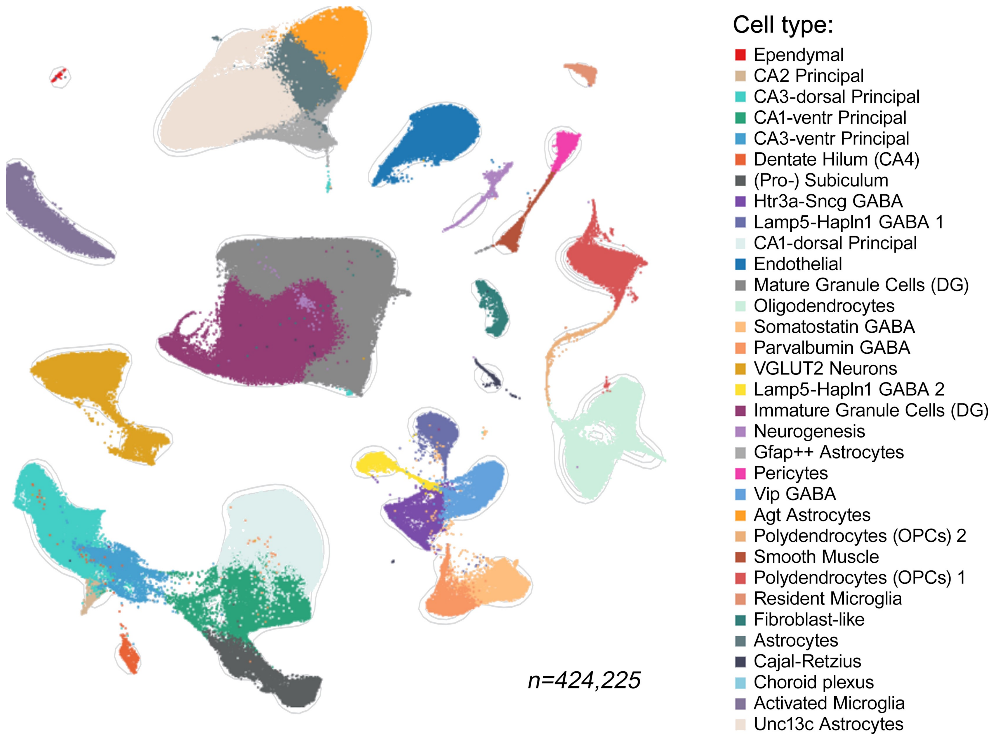

Supplementary Figure 2. UMAP visualization of a comprehensive reference hippocampus atlas (HCA) used for machine learning modelling. The HCA atlas is created by integration of five well-annotated transcriptomic hippocampus datasets including Allen Brain Map, DropViz, and others (see methods). The interactive version is accessible at https://hippo-seq.org/hca, allowing for detailed exploration of hippocampus cell types and investigation of gene expression and co-expression patterns for both atlas and user-defined genes.

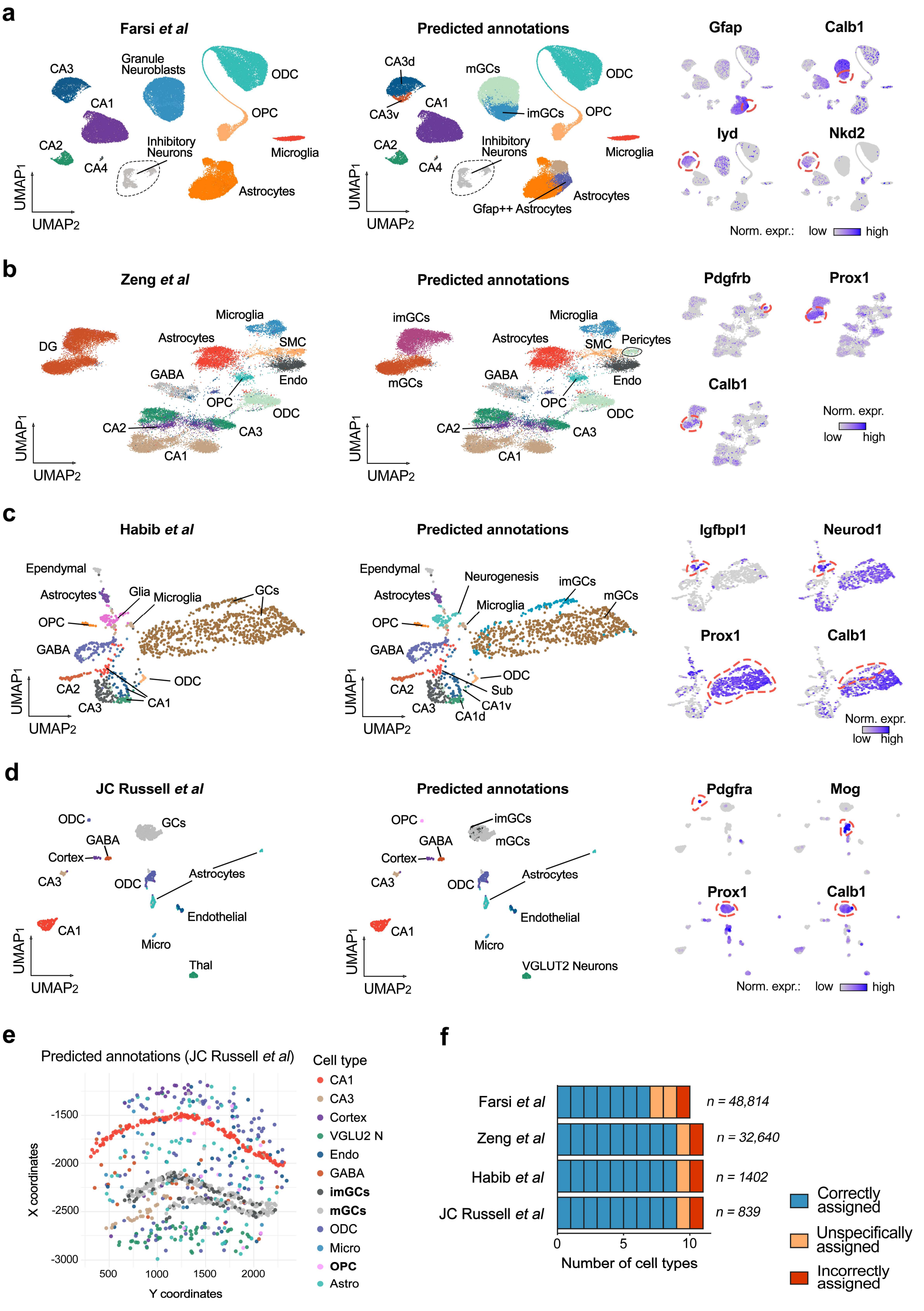

Supplementary Figure 3. Identification of hippocampal cell populations in recently published transcriptomic datasets using novel machine learning-based modeling. (a-d) Left panels: UMAP visualizations of the originally annotated datasets by the authors of the respective studies. Middle panels: UMAP visualizations showing the machine learning-based annotation of the same datasets. Right panels: Expression of supporting markers for the correct reannotation of incorrectly or unspecifically identified cell types. **(e)** Slide-tags-based localization of nuclei to spatial coordinates in the mouse hippocampus, corresponding to the dataset shown in panel d. Cells are colored according to predicted cell type annotations as in the middle panel of d. **(f)** The overall performance of the model across four transcriptomic datasets. The model automatically assigned cell types according to the original studies and reannotated five unspecifically assigned and four incorrectly identified cell types.

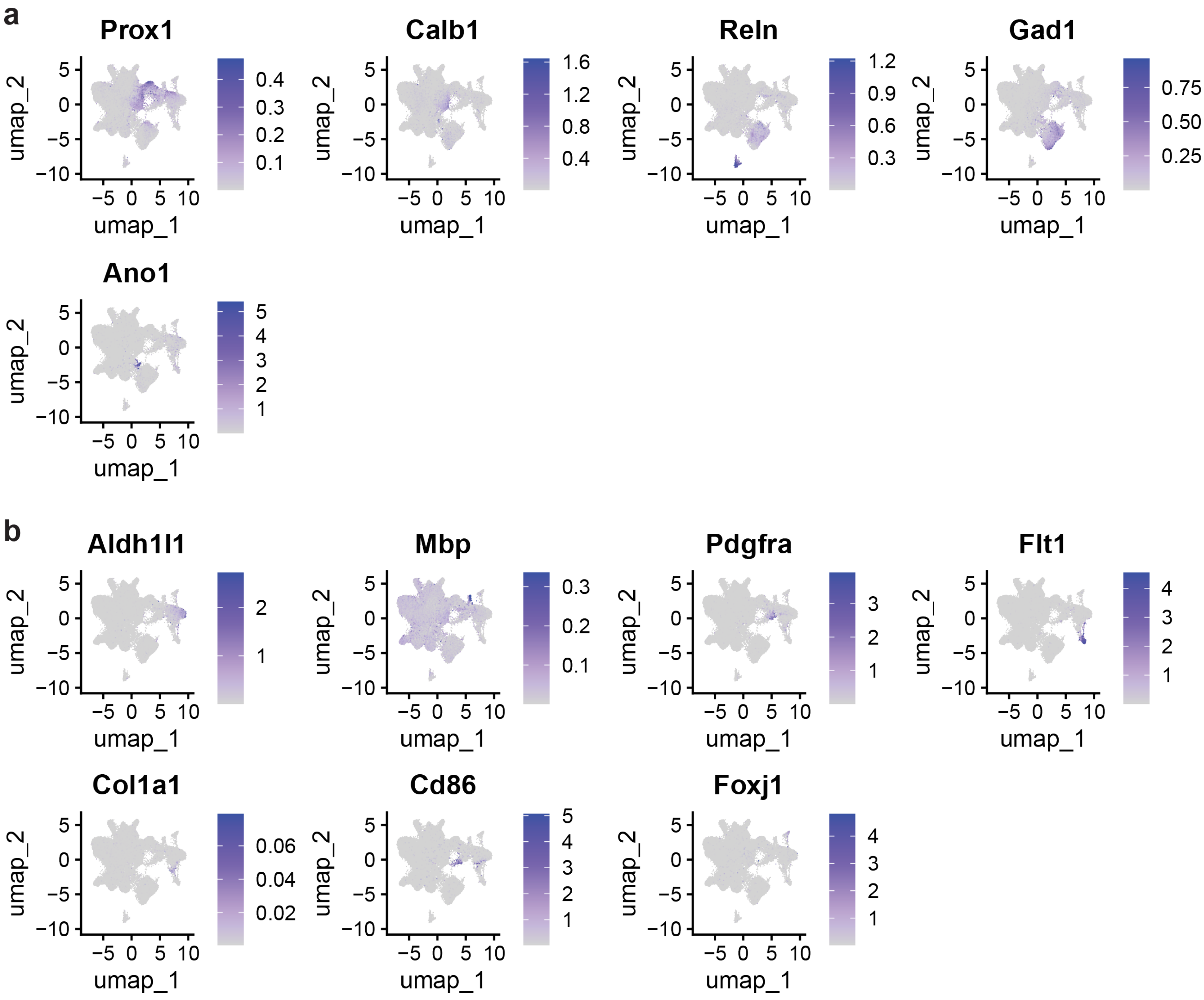

Supplementary Figure 4. Gene expression patterns of key cell type-specific markers in the integrated post-hypoxia-ischemia data. (a) Feature plots showing the expression of neuronal markers: Prox1 and Calb1 (dentate gyrus granule cells), Reln (Cajal-Retzius cells), Gad1 (inhibitory GABA neurons), and Ano1. **(b)** Feature plots displaying non-neuronal cell markers: Aldh1l1 (astrocytes), Mbp (oligodendrocytes), Pdgfra (OPCs), Flt1 (endothelial cells), Col1a1 (fibroblastlike cells), Cd86 (microglia), and Foxj1 (ependymal cells). Color scales indicate normalized expression levels for each gene.

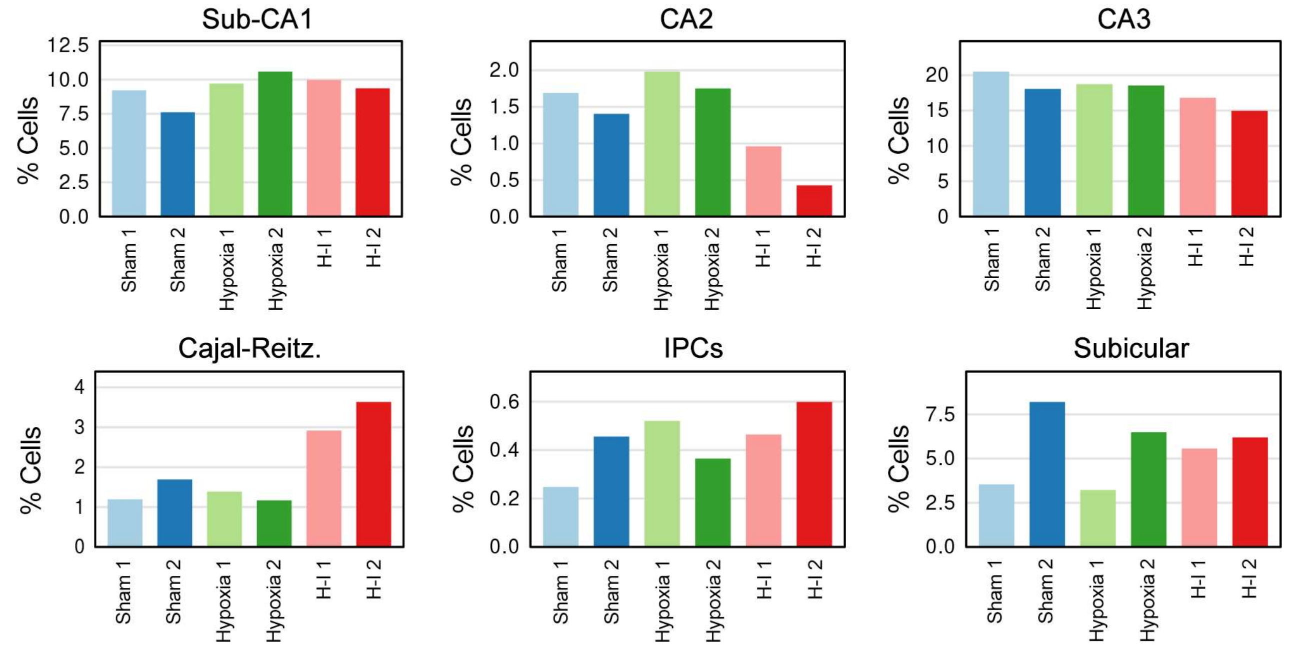

Supplementary Figure 5. Quantitative analysis showing proportional cell changes in various neuronal populations between sham control, post-hypoxia, and post-hypoxia-ischemia conditions in snRNA-seq data.

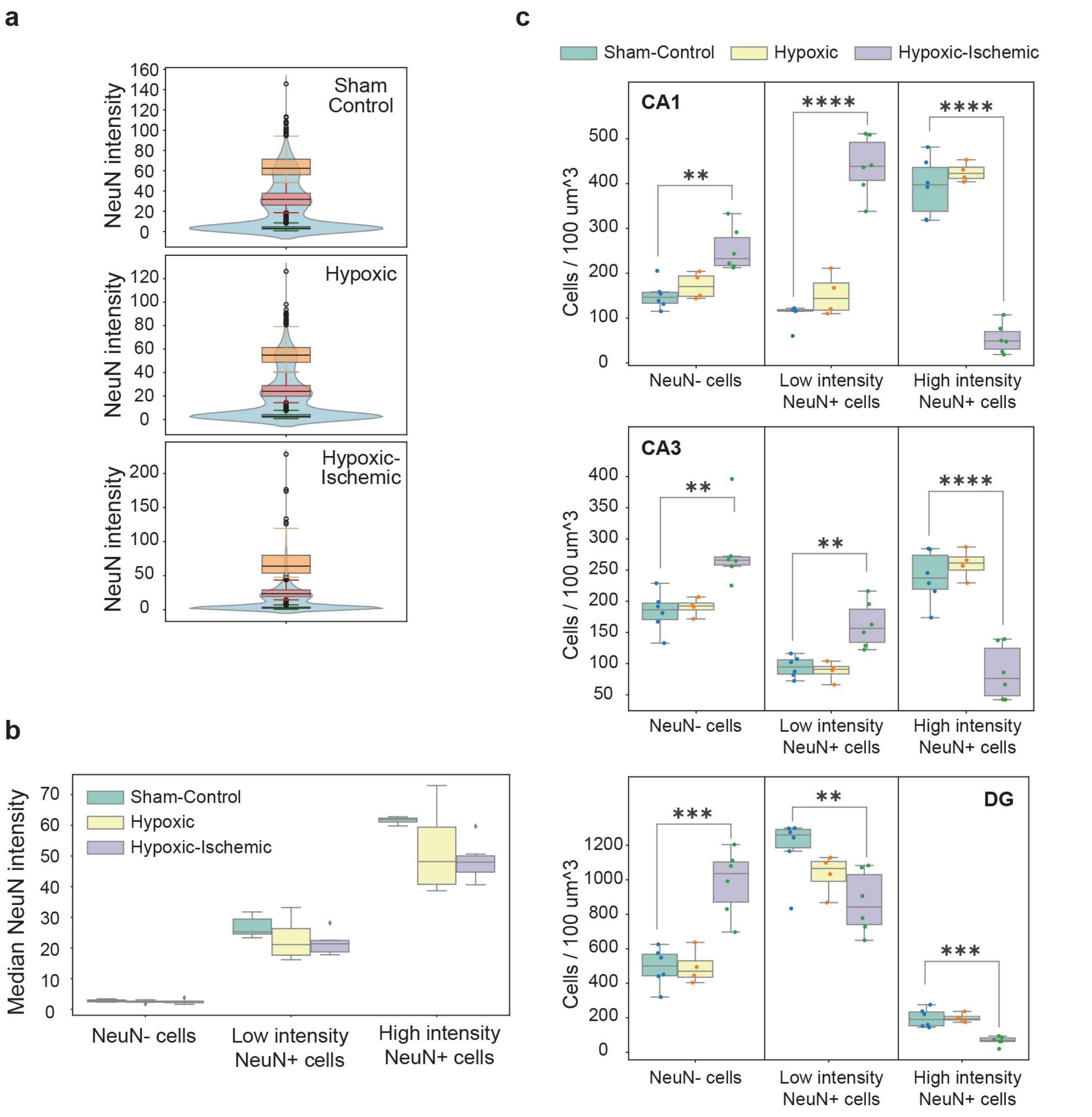

Supplementary Figure 6. Quantitative analysis of NeuN expression in hippocampal subregions under sham, hypoxic, and hypoxic-ischemic conditions. (a) NeuN fluorescent intensity of NeuN in hippocampal nuclei. Fluorescence intensity is reported in arbitrary fluorescence units corresponding to the mean voxel intensity within each nucleus, showing distinct high- and low intensity populations in sham and hypoxic conditions, with predominant low-intensity population in hypoxic-ischemic condition. Boxplots show the clustering of nuclei into three groups by k-means grouping. Outliers deviating more than 1.5 x IQR of each cluster are shown as black circles. 4000-6000 nuclei per condition were used for the analysis. **(b)** Median NeuN intensity of NeuN- as well as low- and high-intensity NeuN+ populations. **(c)** Quantification of NeuN-, low- and high-intensity NeuN+ cell density (cells/100 μm^3) across CA1, CA3, and DG hippocampal regions. Each data point (unfilled black circles) represents a single neuronal nucleus. Data shown as mean ± SEM (sham: n=6 sections/3 animals; hypoxic alone: n=4 sections/3 animals; hypoxic-ischemic: n=6 sections/3 animals). *$p<0.05$, **$p<0.01$, ***$p<0.001$, ****$p<0.0001$.

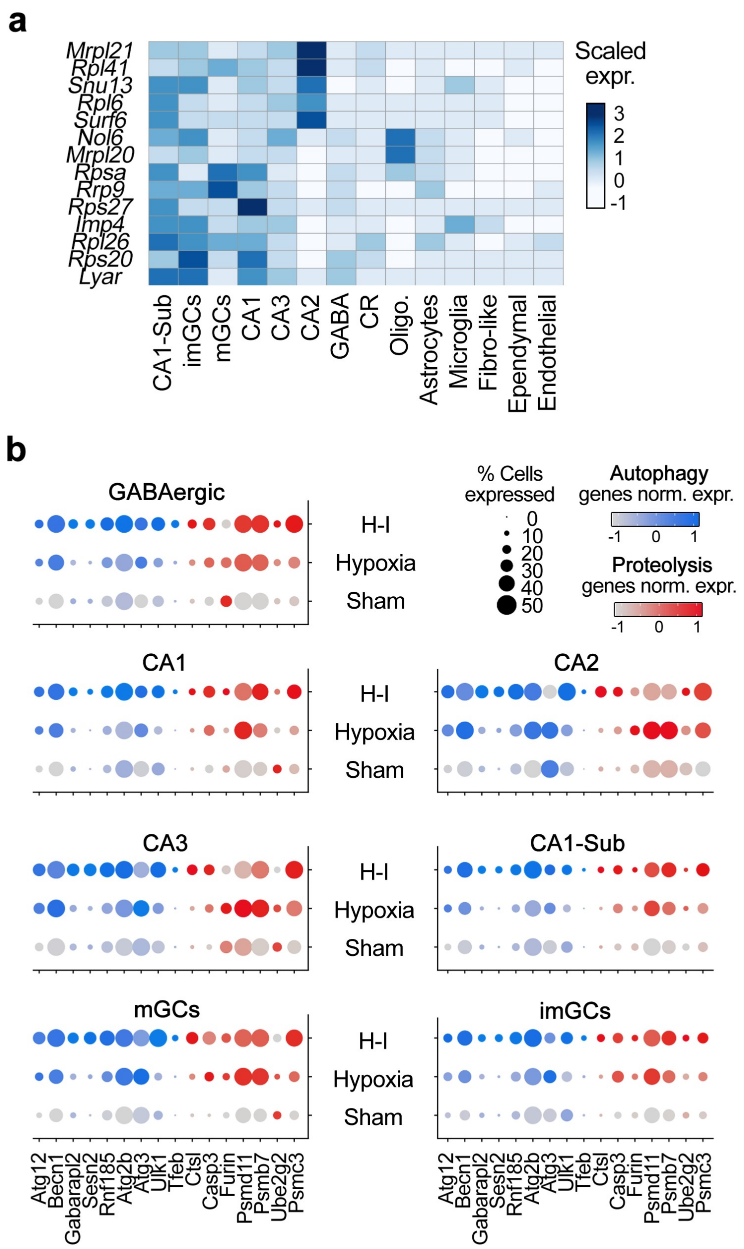

Supplementary Figure 7. Cell-type specific transcriptional responses to hypoxic-ischemic injury. (a) Expression of ribosome biogenesis-related genes across cell populations, showing specific upregulation in CA1-3 and granule cells. **(b)** Expression of autophagy and proteolysis pathway genes across neuronal populations.
